# The nucleolar GTPase nucleostemin-like 1 plays a role in plant growth and senescence by modulating ribosome biogenesis

**DOI:** 10.1093/jxb/erv337

**Published:** 2015-07-10

**Authors:** Young Jeon, Yong-Joon Park, Hui Kyung Cho, Hyun Ju Jung, Tae-Kyu Ahn, Hunseung Kang, Hyun-Sook Pai

**Affiliations:** ^1^Department of Systems Biology, Yonsei University, Seoul 120–749, Korea; ^2^Department of Plant Biotechnology, College of Agriculture and Life Sciences, Chonnam National University, Gwangju 500–757, Korea; ^3^Department of Energy Science, Sungkyunkwan University, Suwon 440–746, Korea

**Keywords:** Delayed 25S rRNA maturation, GTPase activity, nucleolar localization, premature senescence, RNA binding activity, suppressed translation.

## Abstract

Nucleostemin is a nucleolar GTP-binding protein that is involved in stem cell proliferation, embryonic development, and ribosome biogenesis in mammals. Plant nucleostemin-like 1 (NSN1) plays a role in embryogenesis, and apical and floral meristem development. In this study, a nucleolar function of NSN1 in the regulation of ribosome biogenesis was identified. Green fluorescent protein (GFP)-fused NSN1 localized to the nucleolus, which was primarily determined by its N-terminal domain. Recombinant NSN1 and its N-terminal domain (NSN1-N) bound to RNA *in vitro*. Recombinant NSN1 expressed GTPase activity *in vitro*. *NSN1* silencing in *Arabidopsis thaliana* and *Nicotiana benthamiana* led to growth retardation and premature senescence. NSN1 interacted with Pescadillo and EBNA1 binding protein 2 (EBP2), which are nucleolar proteins involved in ribosome biogenesis, and with several ribosomal proteins. NSN1, NSN1-N, and EBP2 co-fractionated primarily with the 60S ribosomal large subunit *in vivo*. Depletion of NSN1 delayed 25S rRNA maturation and biogenesis of the 60S ribosome subunit, and repressed global translation. NSN1-deficient plants exhibited premature leaf senescence, excessive accumulation of reactive oxygen species, and senescence-related gene expression. Taken together, these results suggest that NSN1 plays a crucial role in plant growth and senescence by modulating ribosome biogenesis.

## Highlight

Plant NSN1 has GTPase and RNA-binding activities. In the nucleolus, NSN1 is involved in rRNA maturation and ribosome biogenesis through interaction with PES, EBP2, and several ribosomal proteins.

## Introduction

Ribosome biogenesis is a fundamental process for making protein translation machinery, which is tightly co-ordinated with cell growth and proliferation. Ribosome assembly involves a series of complex processes including synthesis and processing of rRNAs, chemical modification, ordered assembly of the ribosomal components, and maturation. The mechanisms of ribosome biogenesis have been characterized in yeast using genetic and biochemical approaches, which involve four rRNA species, >70 ribosomal proteins, and >150 non-ribosomal factors (Henras *et al.*, 2008; Kressler *et al.*, 2010; Panse and Johnson, 2010; Karbstein, 2011). However, the staggering number of ribosome assembly factors in eukaryotes has hampered full understanding of the ribosomal biogenesis process. Plants encode many evolutionarily conserved ribosome assembly factors, including small nucleolar ribonucleoproteins (SnoRNPs), nucleases, RNA helicases, RNA chaperones, ATPases, and GTPases; however, only a small number of these factors has been functionally analysed (Pendle *et al.*, 2005; Im *et al.*, 2011; Cho *et al.*, 2013; Weis *et al.*, 2014). Recent studies suggest that ribosome assembly factor genes play a role in plant development (Byrne, 2009; Horiguchi *et al.*, 2012).

The RbgA/YlqF/YawG family of GTPases is characterized by a circular permutation of the GTPase signature motifs; this family is broadly conserved in bacteria, archaea, and eukaryotes (Schaefer *et al.*, 2006; Karbstein, 2007; Britton, 2009). In *Bacillus subtilis*, YlqF binds to the premature 50S subunit and mediates a 23S rRNA conformational change, which enables the late-stage assembly of functional 50S ribosomal subunits (Matsuo *et al.*, 2006; Uicker *et al.*, 2006). In yeast, Nug1 and Nug2 GTPases localize to the nucleolus and participate in ribosome biogenesis and export (Bassler *et al.*, 2001, 2006; Saveanu *et al.*, 2001; Matsuo *et al.*, 2014). In mammals, nucleostemin (NS) is preferentially expressed in nucleoli of embryonic stem cells, neuronal stem cells, and several types of cancer cells. The emerging evidence suggests that NS may control cell cycle progression and stem cell proliferation (Ma and Pederson, 2008; Tsai and Meng, 2009; Lo and Lu, 2010). NS inactivation in mice leads to embryo lethality, with the mutant blastocysts exhibiting severe defects in cell proliferation (Beekman *et al.*, 2006), while mouse embryonic fibroblasts of the heterozygous NS-null mice had a lower population growth rate and higher percentages of senescent cells (Zhu *et al.*, 2006). Mammalian NS shuttles between the nucleus and the nucleoplasm in a GTP-driven cycle, and interacts with many proteins residing in the nucleoplasm, such as tumour suppressor p53, mouse double minute 2 (MDM2), and telomeric repeat-binding factor (TRF1) (Tsai and McKay, 2002, 2005; Zhu *et al.*, 2006; Ma and Pederson, 2007, 2008; Dai *et al.*, 2008).

Perturbations in ribosome biogenesis or function ultimately lead to disruptions in cell homeostasis, which is termed nucleolar stress (Dai *et al.*, 2004, 2006; Zhu *et al.*, 2009; James *et al.*, 2014). Nucleolar stress can be induced at multiple steps from pre-rRNA transcription and processing to ribosome maturation and release, and can cause cell cycle arrest, senescence, and apoptosis. Surprisingly, NS knockdown and overexpression both trigger cell cycle arrest in mammalian cells through activation of p53 (Tsai and McKay, 2002; Dai *et al.*, 2008). Tumour suppressor p53 is known to play a key role in cell cycle arrest and apoptosis under unfavourable growth conditions (Haupt *et al.*, 1997; Kubbutat *et al.*, 1997). When NS is deficient, ribosomal proteins L5 and L11 bind to MDM2 and block MDM2-mediated ubiquitination and degradation of p53, leading to cell cycle arrest. Overexpression of NS also leads to G_1_ arrest because overexpressed NS directly interacts with MDM2 and inhibits its E3 ubiquitin ligase activity for p53 (Tsai and McKay, 2002; Dai *et al.*, 2008). These results suggest that a fine balance of NS levels is critical for cellular homeostasis. Recent studies suggest that mammalian NS is important in pre-rRNA processing and ribosome biogenesis (Romanova *et al.*, 2009). NS forms a large protein complex that co-fractionates with the pre-60S ribosomal subunit, and the complex contains ribosome assembly factors such as Pescadillo, DEAD-box helicase 21 (DDX21), EBNA1 binding protein 2 (EBP2), and several ribosomal proteins. Down-regulation of *NS* expression delays processing of 32S pre-rRNA into mature 28S rRNA and suppresses global translation (Romanova *et al.*, 2009). Collectively, these results suggest that mammalian NS may function in responding to nucleolar stress, in addition to its role in ribosome biogenesis under normal growth conditions.

The *Arabidopsis* NS homologue nucleostemin-like 1 (NSN1; At3g07050) has been recently characterized (Wang *et al.*, 2012*a*, *b*). Homozygous *nsn1* mutants were defective in embryogenesis, and leaf and flower development. The *nsn1* mutants exhibited disrupted leaf polarity and meristem-like outgrowths in the adaxial leaf epidermis, which were accompanied by altered expression patterns of the stem cell marker gene *CLAVATA3* (Wang *et al.*, 2012*b*). Termination of the inflorescence meristem and homeotic floral organ transformation were evident in *nsn1* mutant flowers, and the subsequent genetic analyses suggested genetic interaction of *NSN1* with *AGAMOUS* and *APETALA2* (Wang *et al.*, 2012*a*). Consistent with these phenotypes, *NSN1* is highly expressed in developing embryos, shoot apical and floral meristems, and organ primordia, based on *in situ* RNA hybridization (Wang *et al.*, 2012*a*, *b*). The green fluorescent protein (GFP) fusion protein of NSN1 is predominantly localized to the nucleolus in tobacco BY-2 cells (Wang *et al.*, 2012*a*). These results suggest that NSN1 plays a critical role in plant embryogenesis and meristem development. In this study, protein characteristics and nucleolar functions of NSN1 in *Arabidopsis thaliana* and *Nicotiana benthamiana* were investigated.

## Materials and methods

### Plant materials and growth conditions

*Arabidopsis thaliana* (ecotype Columbia-0) and *N. benthamiana* plants were grown in a growth chamber at 22 °C under a 16h light/8h dark cycle. For growth on agar, seeds were surface-sterilized and sown on Petri dishes containing MS medium [Murashige and Skoog salts pH 5.7, 0.35% Phytagel (Sigma), and 2% sucrose] with ethanol [– dexamethasone (DEX)] or with 20 μM DEX.

### Electrophoretic mobility shift assay (EMSA)

To prepare radioactive 16S and 23S rRNA probes, the cDNAs encoding full-length 16S and 23S rRNA were cloned into the pGEM T-easy vector. The constructs were digested with *Bam*HI restriction enzyme, and ^32^P-labelled RNAs were prepared by *in vitro* transcription using T7 RNA polymerase (Promega). For RNA binding assays, the RNA substrates (200ng) were incubated with purified recombinant maltose-binding protein (MBP) fusion proteins (100 pmol) in binding buffer (10mM TRIS-HCl, pH 7.5, 50mM NaCl, 1mM EDTA, 7.4% glycerol) on ice for 30min in the absence or presence of GTP (100 μM). The reaction mixtures were loaded on a 0.8% agarose gel, and RNA bands were visualized by a phosphorimager (GE Healthcare Life Sciences).

### Incorporation of ^35^S-labelled methionine

Using ^35^S-labelled methionine, newly synthesized proteins were detected as described (Ahn *et al.*, 2011; Cho *et al.*, 2013).

### Metabolic labelling of rRNA

Metabolic labelling of rRNA was performed according to Cho *et al.* (2013) with modification. Five seedlings each of wild-type (WT) and DEX-inducible *NSN1* RNAi (RNA interference) lines with or without DEX treatment were incubated overnight with 20 μCi of [α-^32^P]UTP that was diluted in 1ml of MS liquid medium. Total RNA was extracted with a Spectrum™ Plant Total RNA kit (Sigma), according to the manufacturer’s instructions. RNA samples were separated by agarose gel electrophoresis, and the agarose gel was dried and analysed with a phosphorimager (GE Healthcare Life Sciences).

### Sucrose density gradient sedimentation

For polysomal loading analyses, leaf extracts of *Tobacco rattle virus* (TRV), TRV:NbNSN1, and TRV:EBP2 *N. benthamiana* VIGS (virus-induced gene silencing) plants were fractionated through 15–55% sucrose density gradients as described (Ahn *et al.*, 2011). Total proteins were extracted from sucrose density gradient fractions and subjected to SDS–PAGE and immunoblotting with anti-RPL10a antibodies (Santa Cruz Biotechnology).

For co-fractionation analyses, GFP fusion proteins of NSN1, NSN1-N, or EBP2 were expressed in *N. benthamiana* leaves by agroinfiltration, and the leaf extracts were fractionated through 5–35% sucrose density gradients. Proteins extracted from the fractions were separated by SDS–PAGE and subjected to immunoblotting with anti-GFP antibodies (Clontech) and anti-RPL10a antibodies.

Methods describing VIGS; generation of DEX-inducible NSN1 RNAi lines in *Arabidopsis*; *Agrobacterium*-mediated transient expression; real-time quantitative reverse transcription–PCR (RT–PCR); bimolecular fluorescence complementation (BiFC); measurement of *in vivo* H_2_O_2_ levels; immunoblotting; co-immunoprecipitation; purification of recombinant proteins; GTPase assay; pulse amplitude modulation (PAM) fluorometry;and statistical analyses are given in Supplementary Methods S1.

## Results

### Virus-induced gene silencing of *NSN1* in *Nicotiana benthamiana*

**Fig. 1.**
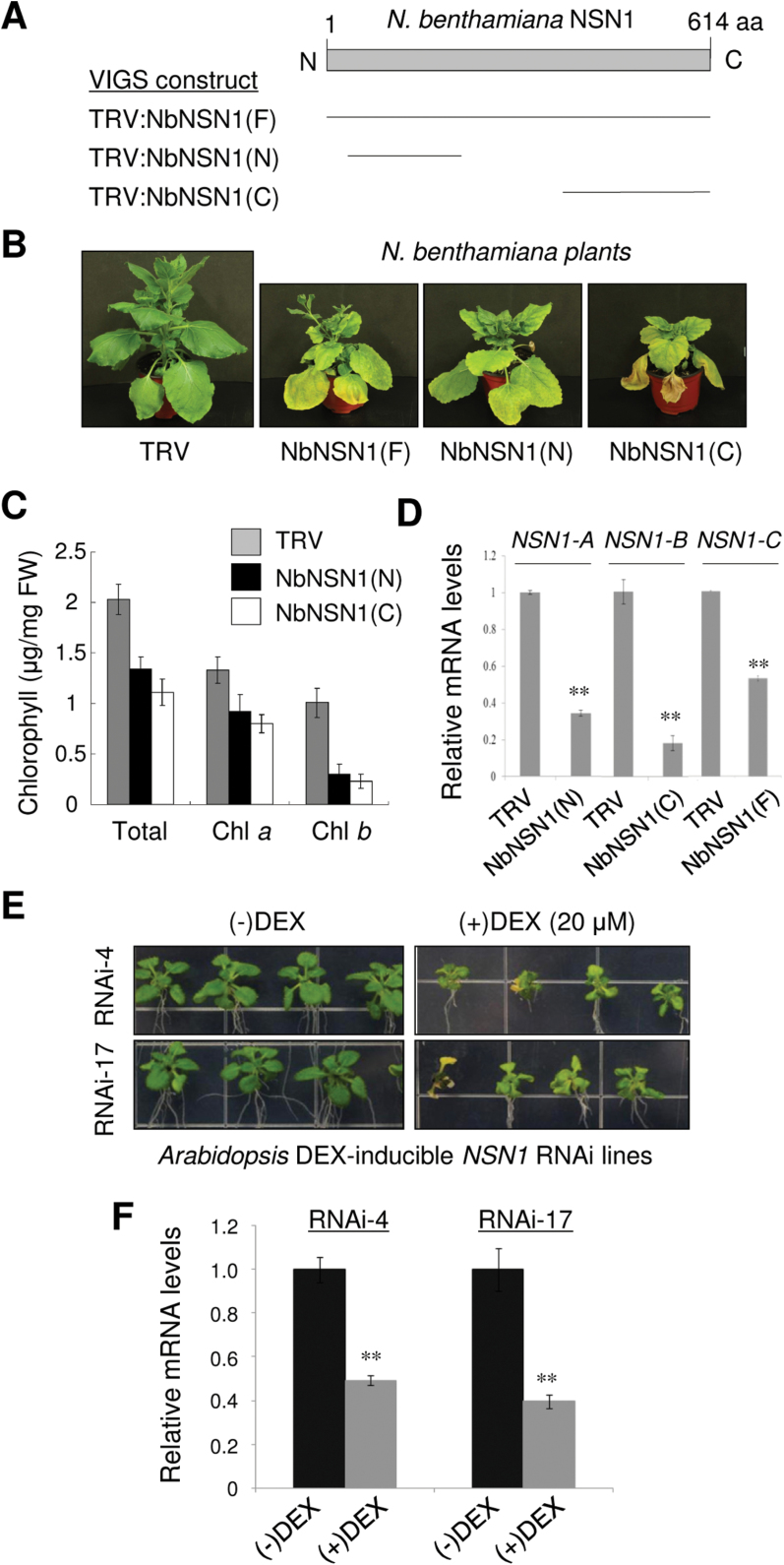
Silencing of *NSN1* using VIGS and DEX-inducible RNAi in *N. benthamiana* and *Arabidopsis*. (A) Schematic of *N. benthamiana NSN1* (*NbNSN1*) structure and three VIGS constructs (F, N, and C) that contain different *NbNSN1* cDNA fragments, as indicated by the bars. (B) Phenotypes of VIGS plants. *NbNSN1* VIGS resulted in growth retardation and premature senescence 20 days after infiltration (DAI), as compared with the control TRV. (C) Quantification of total chlorophyll, chlorophyll *a*, and chlorophyll *b* contents in TRV and TRV:NbNSN1 plants (20 DAI). The fourth leaf above the infiltrated leaf was used for the analysis. (D) Real-time quantitative RT–PCR analysis of *NbNSN1* transcript levels in TRV:NbNSN1(N), TRV:NbNSN1(C), and TRV:NbNSN1(F) plants (14 DAI) using *NSN1-A*, *NSN1-B*, and *NSN1-C* primers, respectively. The fourth leaf above the infiltrated leaf was used for the analysis. The α-tubulin mRNA level was used as control. Data represent the mean ±SD of three replicates per experiment; *P≤0.05; **P≤0.01. (E) Growth retardation and premature senescence in *Arabidopsis* DEX-inducible *NSN1* RNAi lines (#4 and #17) in response to DEX treatment. Seedlings were grown for 18 d on media that contained either ethanol (–DEX) or 20 μM DEX (+DEX). (F) Real-time quantitative RT–PCR analysis of *NSN1* transcript levels in the RNAi lines (#4 and #17) grown for 2 weeks on (–)DEX or (+)DEX media. RNA was isolated from the whole seedlings. The *UBC10* mRNA level was used as control. (This figure is available in colour at *JXB* online.)

Multispecies sequence alignment revealed that plant NSN1 proteins are homologous to yeast Nug1 and human NS, particularly in the N-terminal region and the circularly permutated GTP-binding motifs (Supplementary Fig. S1 available at *JXB* online). According to the Genevestigator program (https://www.genevestigator.com/), *Arabidopsis NSN1* (At3g07050) is constitutively expressed in various tissues, and exhibits high transcript levels throughout plant development (Supplementary Fig. S2A, B). To determine the *in vivo* effects of NSN1 deficiency in *N. benthamiana* and *A. thaliana*, VIGS and DEX-inducible RNAi were performed using the protocol described in Supplementary Methods S1. For VIGS, three different *N. benthamiana NSN1* (*NbNSN1*) cDNA fragments were cloned; these fragments were designated NbNSN1(F), NbNSN1(N), and NbNSN1(C), which contained the 1842bp full-length coding region, a 489bp N-terminal region, and a 638bp C-terminal region of the *NbNSN1* cDNA, respectively. The three fragments were cloned into the TRV-based VIGS vector, pTV00, to create TRV:NbNSN1(F), TRV:NbNSN1(N), and TRV:NbNSN1(C) (Fig. 1A). These vectors were transformed into *Agrobacterium tumefaciens*, and *N. benthamiana* plants were infiltrated with the *Agrobacterium* transformants. VIGS using these TRV:NbNSN1 constructs resulted in growth retardation and premature senescence with reduced chlorophyll contents in leaves compared with the TRV control (Fig. 1B, C; Supplementary Fig. S3). Real-time quantitative RT–PCR revealed significantly lower levels of endogenous *NbNSN1* transcripts in leaves of TRV:NbNSN1(N), TRV:NbNSN1(C), and TRV:NbNSN1(F) VIGS plants, indicating silencing of *NSN1* (Fig. 1D; Supplementary Table S1).

### Generation of dexamethasone-inducible *NSN1* RNAi lines in *Arabidopsis*

Next DEX-inducible *NSN1* RNAi lines were generated in *Arabidopsis* (Fig. 1E, F). Transgenic *Arabidopsis* plants (ecotype Col-0) carried an *NSN1* RNAi construct containing an inverted repeat of the 318bp C-terminal cDNA fragment under the control of a DEX-inducible transcription system. The DEX-inducible *NSN1* RNAi lines were grown on MS medium containing 20 μM DEX solubilized in ethanol (+DEX) or ethanol alone (–DEX) as a control. Seedlings of two independent *NSN1* RNAi lines (#4 and #17) exhibited significantly retarded shoot and root growth, and early onset of senescence on DEX-containing media (Fig. 1E). The effect of RNAi on *NSN1* mRNA levels in seedlings was determined by real-time quantitative RT–PCR using *UBC10* mRNA as a control (Fig. 1F). Seedlings of lines RNAi-4 and RNAi-17 grown on DEX-containing media had reduced *NSN1* transcript levels compared with seedlings grown without DEX.

### Subcellular localization of NSN1 and its deletion mutants

**Fig. 2.**
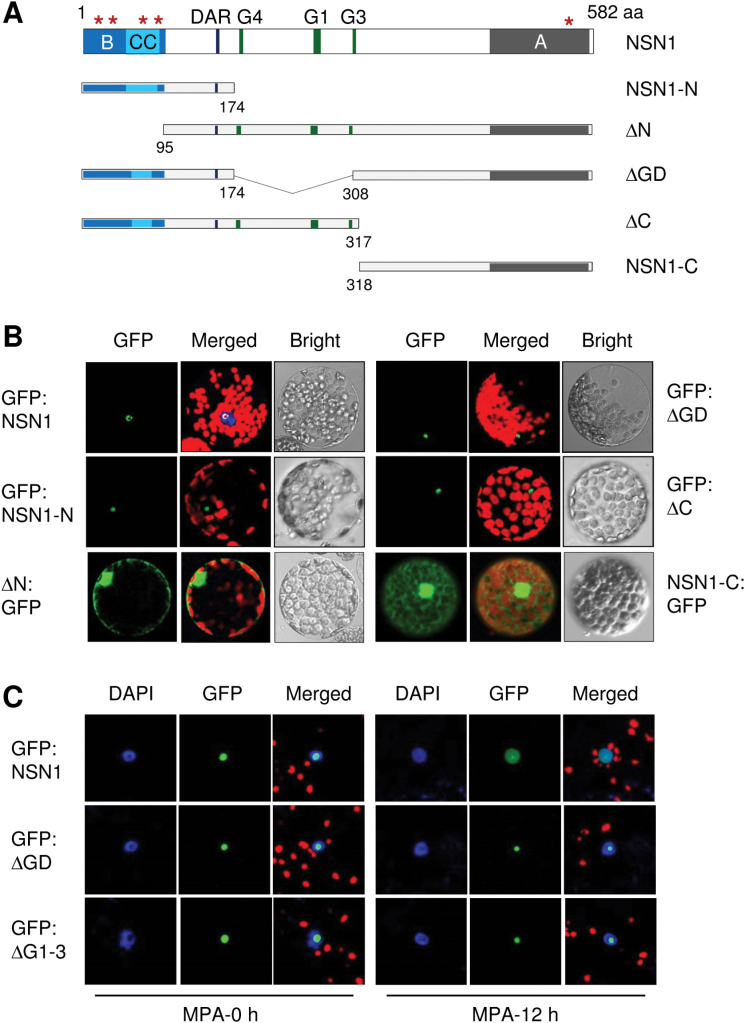
Subcellular localization of *Arabidopsis* NSN1 and its deletion mutants. (A) Schematic of NSN1 and NSN1 deletion mutants. B, basic domain (amino acids 1–93); CC, coiled-coil domain (amino acids58–80); DAR, the DAR motif (amino acids 145–147), G4–G3, GTP-binding motifs (amino acids 175–307); and A, acidic domain (amino acids 471–570). Amino acid (aa) residues at deletion points are marked. Asterisks indicate nuclear localization signals. (B) Subcellular localization of NSN1 and NSN1 deletion mutants using GFP fusion. GFP fusion proteins were expressed in *N. benthamiana* leaves via agroinfiltration, and protoplasts prepared from the infiltrated leaves were observed by confocal laser scanning microscopy for GFP fluorescence and chlorophyll autofluorescence. Protoplasts expressing GFP:NSN1 were stained with 4′,6-diamidino-2-phenylindole (DAPI) to visualize the nucleus. (C) Effects of mycophenolic acid (MPA) on the localization of NSN1 and NSN1 deletion mutants. *Nictiana benthamiana* leaves were agroinfiltrated with GFP fusion constructs and then treated with MPA (20 μM) for 12h. Nuclei were visualized by DAPI staining. (This figure is available in colour at *JXB* online.)

NSN1 contains a predicted basic domain (B), a coiled-coil domain (CC), the DAR motif, the GTP-binding motifs (G4, G1, and G3), and an acidic domain (A) (Fig. 2A; Supplementary Fig. S1 at *JXB* online). Multiple nuclear localization signals are located in both the N- and C-terminal regions, which are marked with asterisks. To determine the subcellular localization of NSN1, GFP fusion proteins of *Arabidopsis* NSN1 and NSN1-deletion mutants were expressed in *N. benthamiana* leaves via agroinfiltration. GFP was fused to the N-termini of NSN1 and its derivatives. However, the N-terminal deletion form (∆N) and the C-terminal region (NSN1-C) were more stable when fused to GFP via their C-termini. Confocal laser scanning microscopy of leaf protoplasts revealed that GFP:NSN1 was predominantly localized to the nucleolus (Fig. 2B; Supplementary Fig. S4). Deletion of the GTP-binding motifs G4–G3 (∆GD) or the C-terminal region (∆C) did not affect NSN1 nucleolar localization (Fig. 2B). However, deletion of the N-terminal 94 amino acids (∆N) resulted in NSN1 distribution in the nucleus and the cytosol (Fig. 2B; Supplementary Fig. S4). The deletion mutant lacking the N-terminal 174 amino acids was not stably expressed in *N. bethamiana* leaves, regardless of the position of GFP tagging. The N-terminus (NSN1-N; amino acid residues 1–174) was sufficient to target the GFP fusion protein to the nucleolus. The GFP fusion protein containing the NSN1 C-terminal region (NSN1-C; amino acids 318–582) localized to both the nucleus and the cytosol (Fig. 2B; Supplementary Fig. S5). This result suggests that the NSN1 N-terminal region is crucial for NSN1 nucleolar localization.

Next it was tested whether GTP depletion within a cell affects the nucleolar localization of either NSN1 or NSN1-deletion mutants lacking the GTP-binding motifs (Fig. 2C). *Nicotiana benthamiana* leaves were agroinfiltrated to express GFP fusion proteins of NSN1 and NSN1-deletion mutants, and then treated with mycophenolic acid (MPA). MPA inhibits inosine monophosphate dehydrogenase (IMPDH), the rate-limiting enzyme for the *de novo* synthesis of guanine nucleotides (Tsai and McKay, 2005; Huang *et al.*, 2008). Confocal microscopy revealed that NSN1 translocated from the nucleolus to the nucleoplasm after 12h of MPA treatment (Fig. 2C). However, the NSN1 mutants ∆GD and ∆G1–3 (deletion of the G1–G3 motifs), did not undergo translocation; their GFP fusion proteins remained in the nucleolus after MPA treatment. These results suggest that GTP depletion mediated by IMPDH inhibition causes repartitioning of NSN1 into the nucleoplasm, and this translocation requires intact GTP-binding motifs of NSN1.

### RNA binding activity of NSN1

**Fig. 3.**
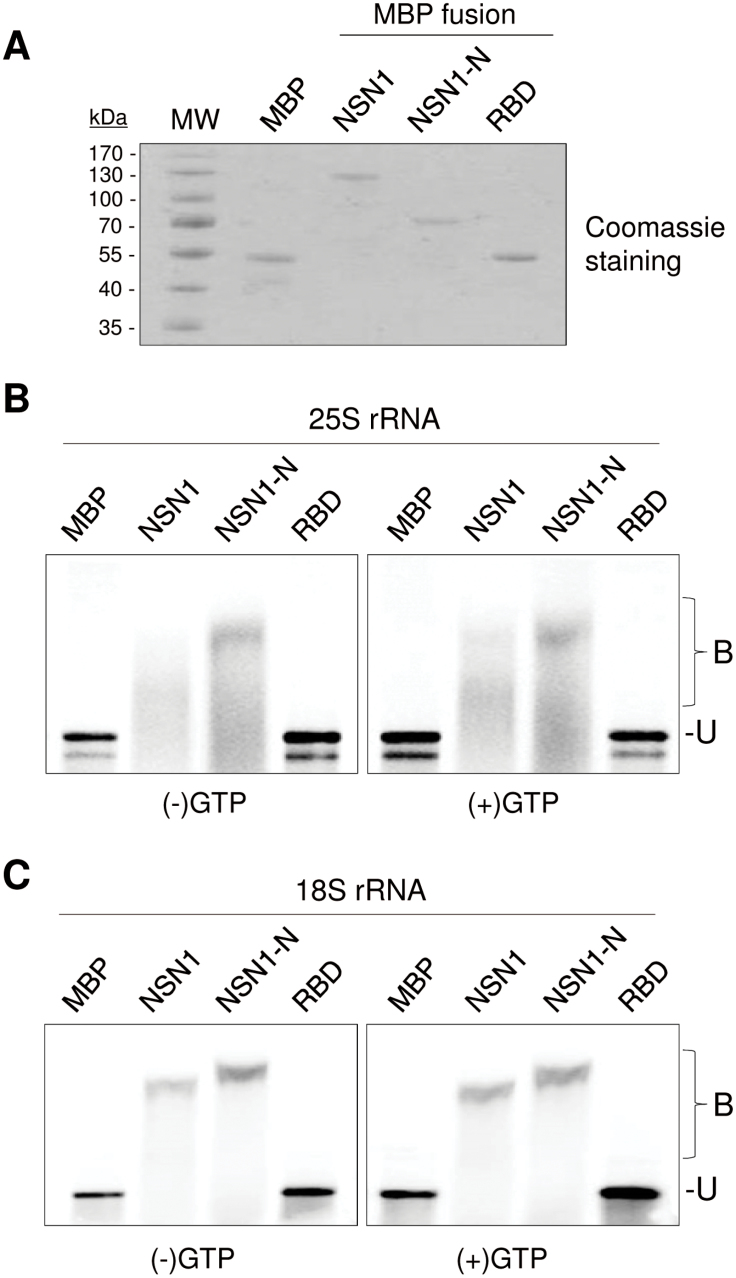
Gel mobility shift assay to detect RNA binding activity of NSN1. (A) Maltose-binding protein (MBP)-fused NSN1 and NSN1 derivatives were purified from *E. coli*, and the eluted proteins were visualized by Coomassie blue staining. NSN1-N, N-terminal domain of NSN1 (amino acids 1–174); RBD, a putative RNA-binding domain of NSN1 (amino acids 374–400). Size markers are indicated. (B, C) MBP, MBP:NSN1, MBP:NSN1-N, and MBP:RBD fusion proteins (100 pmol) were incubated with 200ng of radiolabelled 25S rRNA (B) or 18S rRNA (C) with or without GTP (100 μM). Bound (B) and unbound (U) RNAs were resolved on an agarose gel and visualized by phosphorimaging.

It has been reported that the ribosome assembly GTPases contain RNA-binding domains in addition to the GTPase domain; yeast Nug1 binds directly to 5S rRNA and tRNA via its N-terminal domain (Bassler *et al.*, 2006; Karbstein, 2007). To determine if NSN1 binds RNA, recombinant proteins of full-length NSN1 and the NSN1 N-terminal domain (NSN1-N; amino acids 1–174) fused to MBP were prepared. The RNA binding activity of a region (residues 374–400) that was previously annotated as a putative RNA-binding domain (RBD; Wang *et al.*, 2012*a*) was also tested. The corresponding cDNA fragments were cloned into the pMAL vector, expressed in *Escherichia coli*, and recombinant MBP fusion proteins were affinity-purified using the N-terminal MBP tag (Fig. 3A). The binding of these recombinant proteins to 25S rRNA as an RNA substrate was determined using EMSAs. ^32^P-labeled 25S rRNA synthesized *in vitro* was incubated with MBP:NSN1, MBP:NSN1-N, MBP:RBD, and MBP in the absence or presence of 100 μM GTP, and RNA–protein complexes were resolved by agarose gel electrophoresis. Both MBP:NSN1 and MBP:NSN1-N readily formed stable RNA–protein complexes regardless of the GTP status, whereas neither MBP:RBD nor MBP complexed with RNA (Fig. 3B). MBP:NSN1 and MBP:NSN1-N also formed RNA–protein complexes with ^32^P-labelled 18S rRNA with or without GTP, whereas MBP:RBD or MBP did not (Fig. 3C). These results indicate that NSN1 has an RNA-binding activity, and the NSN1 N-terminal domain contributes to the binding.

### GTPase activity of NSN1

The GTPase activity of the recombinant NSN1 protein fused to MBP was then measured (Table 1). GTP hydrolysis assay was performed to determine the turnover number (*k*_cat_) of MBP:NSN1, and the value was compared with those of NSN1-related GTPases. At steady state, the *k*_cat_ value was 5.46h^–1^ for NSN1; 5.22h^–1^ for *Arabidopsis* Nug2; 7.2h^–1^ for rice Nug2; 6.6h^–1^ for *Saccharomyces cerevisiae* Nug1; and 7.8h^–1^ for *E. coli* YjeQ (Table 1). Thus, the GTPase activity of NSN1 was comparable with those of related eukaryotic and prokaryotic GTPases.

**Table 1.** GTPase activity of NSN1 in comparison with those of related GTPases

Protein	*k*_*cat*_ (h^–1^)	References
NSN1	5.46±0.96	This study
AtNug2 (*Arabidopsis*)	5.22±0.12	Im *et al.* (2011)
OsNug2 (rice)	7.2±0.42	Im *et al.* (2011)
Nug1 (*S. cerevisiae*)	6.6±0.6	Bassler *et al.* (2006)
YjeQ (*E. coli*)	7.8	Daigle *et al.* (2002)

GTPase assay was performed as described in the Materials and methods.

Data represent the mean ±standard deviation (SD) of three replicates per experiment.

### NSN1 interactions with PES, EBP2, and several ribosomal proteins

**Fig. 4.**
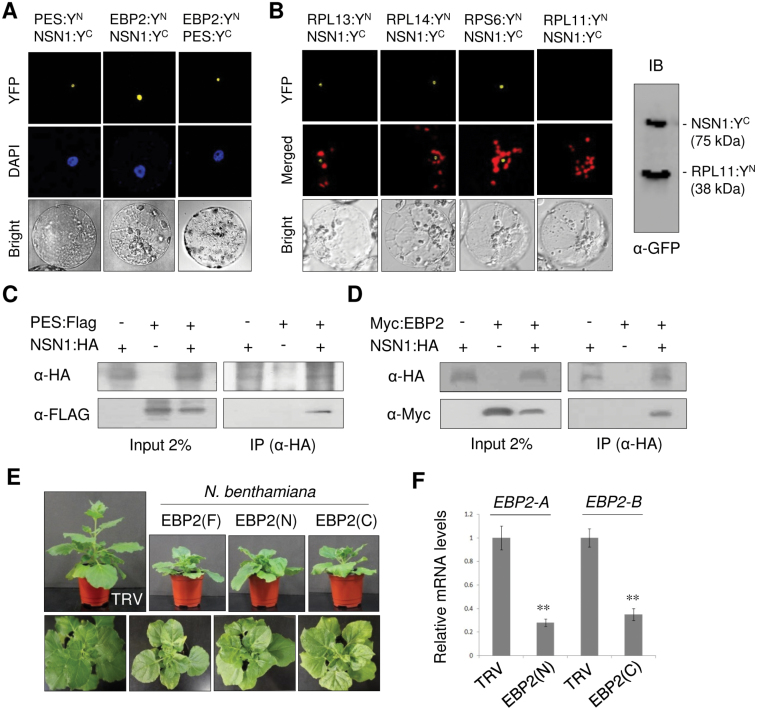
Analyses of protein interactions of NSN1. (A) BiFC analyses of NSN1 interaction with PES and EBP2. Proteins were co-expressed as either YFP^N^ (Y^N^) or YFP^C^ (Y^C^) fusion proteins in *N. benthamiana* leaves using agroinfiltration. Protoplasts were prepared from the infiltrated leaves and observed for YFP fluorescence after brief staining with DAPI to visualize nuclei. (B) BiFC analyses of NSN1 interaction with ribosomal proteins. Left, confocal microscopy; right, immunoblot analysis (IB). (C, D) Co-immunoprecipitation. Protein extracts were prepared from *N. benthamiana* leaves co-expressing PES:Flag and NSN1:HA (C) or Myc:EBP2 and NSN1:HA (D) fusion proteins. Extracts were subjected to immunoprecipitation (IP) with anti-HA antibody, and co-immunoprecipitated proteins were detected by immunoblotting with anti-Flag and anti-Myc antibodies. (E) VIGS phenotypes of *EBP2* using three different *EBP2* constructs in *N. benthamiana* at 14 DAI. (F) Real-time quantitative RT–PCR analysis of *EBP2* transcript levels in TRV:EBP2(N) and TRV:EBP2(C) plants (14 DAI) using *EBP2-A* and *EBP2-B* primers. The fourth leaf above the infiltrated leaf was used for the analysis. The β-tubulin mRNA level was used as control. Data represent the mean ±SD of three replicates per experiment; *P≤0.05; **P≤0.01. (This figure is available in colour at *JXB* online.)

Mammalian NS forms a large protein complex that contains Pescadillo, EBP2, DDX21, and a subset of ribosomal proteins (Romanova *et al.*, 2009). BiFC was used to test if NSN1 interacts with *Arabidopsis* Pescadillo (PES) and EBP2 *in vivo* using (Fig. 4A). BiFC has been widely used for visualization of protein–protein interactions in living cells (Walter *et al.*, 2004). Plant PES is a nucleolar protein that plays a crucial role in biogenesis of the 60S ribosomal large subunit through a functional link with BOP1 and WDR12 (Cho *et al.*, 2013). Combinations of proteins were co-expressed as N- and a C-terminal yellow fluorescent protein fusion proteins (YFP^N^ and YFP^C^) in *N. benthamiana* leaves by agroinfiltration. After 48h, protoplasts were prepared from the infiltrated leaves and observed with confocal laser scanning microscopy. All combinations of fusion protein expression resulted in strong nucleolar YFP fluorescence, indicating that NSN1, PES, and EBP2 interact with each other in the nucleolus (Fig. 4A). The N-terminal domain of NSN1 (NSN1-N; amino acids 1–174) was sufficient for nucleolar interaction with PES and EBP2, whereas the deletion of the NSN1 N-terminal 94 amino acids (∆N) caused protein interactions in both the nucleolus and the nucleoplasm (Supplementary Fig. S6 at *JXB* online). A deletion of the GTP-binding motifs (∆GD) or the NSN1 C-terminal domain (∆C) did not affect the protein interactions in the nucleolus. These results suggest that the NSN1 N-terminal domain plays a role in the interactions of NSN1 with PES and EBP2, but other domains of NSN1 also contribute to the protein interactions. Next, BiFC interactions between NSN1 and PES mutants were examined (Supplementary Fig. S7). NSN1 interacted with the N-terminal PES-N domain in the nucleolus, but with ∆PES-N (a PES mutant lacking the PES-N domain) in the nucleus. Previously, GFP-fused ∆PES-N exhibited a diffuse nucleoplasmic distribution (Cho *et al.*, 2013). These results suggest that PES interaction with NSN1 involves the PES-N domain as well as other regions of PES. BiFC analyses also revealed an interaction of NSN1 with ribosomal proteins RPL13, RPL14, and RPS6, but not with RPL11 (Fig. 4B, left), despite the high expression of both NSN1:YFP^C^ and RPL11:YFP^N^ in the infiltrated leaves (Fig. 4B, right).

Next, co-immunoprecipitation assays were performed. Flag-fused PES (PES:Flag) and haemagglutinin-fused NSN1 (NSN1:HA) (Fig. 4C), or Myc-fused EBP2 (Myc-EBP2) and NSN1:HA (Fig. 4D) were expressed together in *N. benthamiana* leaves by agroinfiltration. NSN1:HA proteins were immunoprecipitated from cell extracts of infiltrated leaves with anti-HA antibodies. Following immunoprecipitation, immunoblotting was first performed with anti-HA antibodies to detect immunoprecipitated NSN1:HA, and then with anti-Flag antibodies to detect PES:Flag as a co-immunoprecipitant (Fig. 4C), or with anti-Myc antibodies to detect Myc:EBP2 as a co-immunoprecipitant (Fig. 4D). The results suggested that PES:Flag and Myc:EBP2 were co-immunoprecipitated with NSN1:HA. However, co-immunoprecipitation did not occur when NSN1:HA was expressed alone. These results further support NSN1 interactions with PES and EBP2 *in vivo*.

VIGS phenotypes resulting from silencing of *N. benthamiana EBP2* (Fig. 4E) were examined in comparison with those of *NbNSN1* (Fig. 1B). The VIGS constructs TRV:EBP2(F), TRV:EBP2(N), and TRV:EBP2(C) contained a 903bp full-length coding region, a 432bp N-terminal region, and a 468bp C-terminal region of *EBP2* cDNA, respectively. VIGS of *EBP2* in *N. benthamiana* resulted in growth retardation similar to that induced by *NbNSN1* VIGS, accompanied by mild leaf yellowing. Real-time quantitative RT–PCR analyses detected lower levels of endogenous *EBP2* transcripts in TRV:EBP2(N) and TRV:EBP2(C) leaves, indicating *EBP2* silencing in the VIGS plants (Fig. 4F).

### Ribosome association of NSN1

**Fig. 5.**
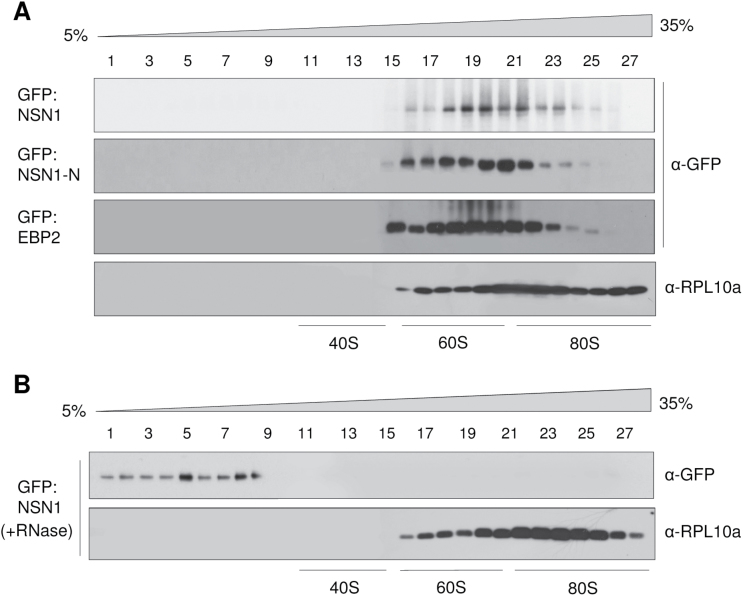
Co-fractionation of NSN1 with ribosome subunits. (A) GFP fusion proteins of NSN1, NSN1-N, and EBP2 were expressed in *N. benthamiana* leaves by agroinfiltration. After sedimentation of ribosomes through a sucrose density gradient, the fractions were analysed by immunoblotting with anti-GFP and anti-ribosomal protein L10a (RPL10a) antibodies. Lanes 1–27 indicate the gradient fractions from the top (5%) to the bottom (35%). Positions of 40S small subunits, 60S large subunits, and 80S monosomes are indicated. RPL10a is present in 60S large subunits and 80S monosomes. (B) NSN1:GFP was expressed in *N. benthamiana* leaves by agroinfiltration. Ribosomal fractions were briefly treated with RNase A before sedimentation through a sucrose density gradient.

To investigate the association of NSN1 and EBP2 with ribosomes, NSN1, its N-terminal domain (NSN1-N), and EBP2 were expressed as GFP fusion proteins in *N. benthamiana* leaves by agroinfiltration. Then, leaf cells expressing these proteins were fractionated on a sucrose density gradient. After ultracentrifugation, fractions were collected, and immunoblot analysis was performed with anti-GFP antibodies (Fig. 5A). As a control for fractionation, another immunoblot analysis was performed with antibodies against the 60S ribosomal protein L10a (RPL10a). GFP:NSN1, GFP:NSN1-N, and GFP:EBP2 were enriched in fractions that contained 60S large subunits and 80S monosomes, suggesting that NSN1 and EBP2 may associate with ribosomes (Fig. 5A). These results also suggest that the NSN1 N-terminal region is important for NSN1 association with ribosomes.

Because NSN1 can bind to RNA, it was examined whether NSN1 binds ribosomes via an RNA tether (Fig. 5B). A brief RNase treatment of the ribosomal fractions inhibited NSN1 incorporation into the ribosomal fractions, suggesting that its interaction with rRNA contributes to NSN1 association with the ribosome subunits. RPL10a incorporation was not affected by the RNase treatment, suggesting more stable association.

### Reduced global translation activity in *NSN1*-silenced plants

**Fig. 6.**
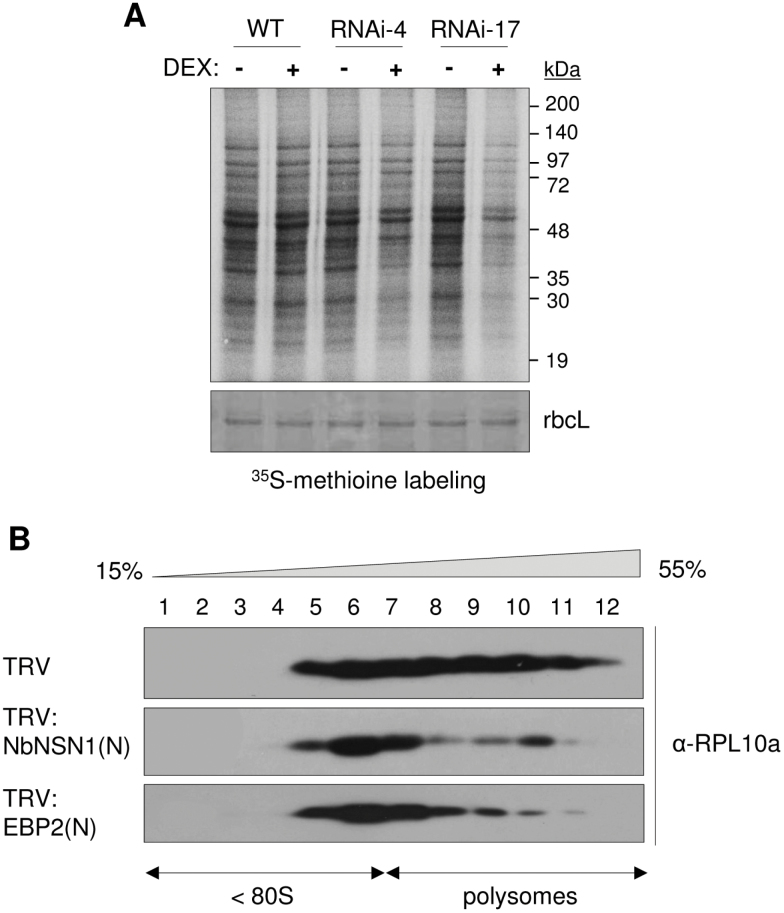
Reduced global translation in NSN1-deficient plants. (A) [^35^S]Methionine labelling. After [^35^S]methionine labelling of *Arabidopsis* RNAi seedlings, total protein extracts were separated by SDS–PAGE, and the gels were dried and analysed with a phosphorimager. A duplicate gel was stained with Coomassie blue to show the Rubisco large subunit (rbcL) as loading control. Whole seedlings grown for 7–8 d on (–)DEX or (+)DEX media were used for the analyses. (B) Polysome loading of RPL10a. Sucrose density gradient fractions from *N. benthamiana* TRV, TRV:NbNSN1(N), and TRV:EBP2(N) VIGS plants (14 DAI) were analysed by immunoblotting with anti-RPL10a antibody. The fourth to fifth leaf above the infiltrated leaf was used for the analysis. Positions of polysomes are indicated. Lanes 1–10 indicate the gradient fractions from the top (15%) to the bottom (55%).

Cellular protein translation activity was examined using [^35^S]methionine labelling in WT *Arabidopsis* and the DEX-inducible *NSN1* RNAi (#4 and #17) lines (Fig. 6A). Seedlings were grown on media with or without DEX for 7–8 d, briefly incubated with [^35^S]methionine, and proteins were extracted. The radioactive protein profiles were obtained using SDS–PAGE of the protein extracts and phosphorimager analysis. Nascent protein synthesis in DEX-treated *NSN1* RNAi (#4 and #17) lines was reduced compared with (–)DEX RNAi seedlings or DEX-treated WT seedlings. This result suggests that NSN1 deficiency leads to a reduction in protein synthesis (Fig. 6A).

The global translation activity in NSN1-deficient *N. benthamiana* plants was also examined by assessing polysomal loading of the 60S ribosomal protein RPL10a. Leaf extracts from TRV, TRV:NbNSN1(N), and TRV:EBP2(N) VIGS plants were sedimented through a 15–55% sucrose density gradient. Fractions were collected, and immunoblot analysis of each fraction was performed using anti-RPL10a antibody. TRV:NbNSN1(N) and TRV:EBP2(N) plants had less RPL10a in the polysome fractions than the TRV control, suggesting that NSN1- and EBP2-deficient leaf cells contained fewer polysomes and lower translation activity (Fig. 6B). Immunoblotting using anti-RPL10a antibody revealed that RPL10a protein levels in TRV:NbNSN1 and TRV:EBP2 leaf cells were similar to the TRV control level (Supplementary Fig. S8 at *JXB* online).

### Delayed 25S rRNA maturation and 60S ribosome biogenesis in NSN1-silenced plants

**Fig. 7.**
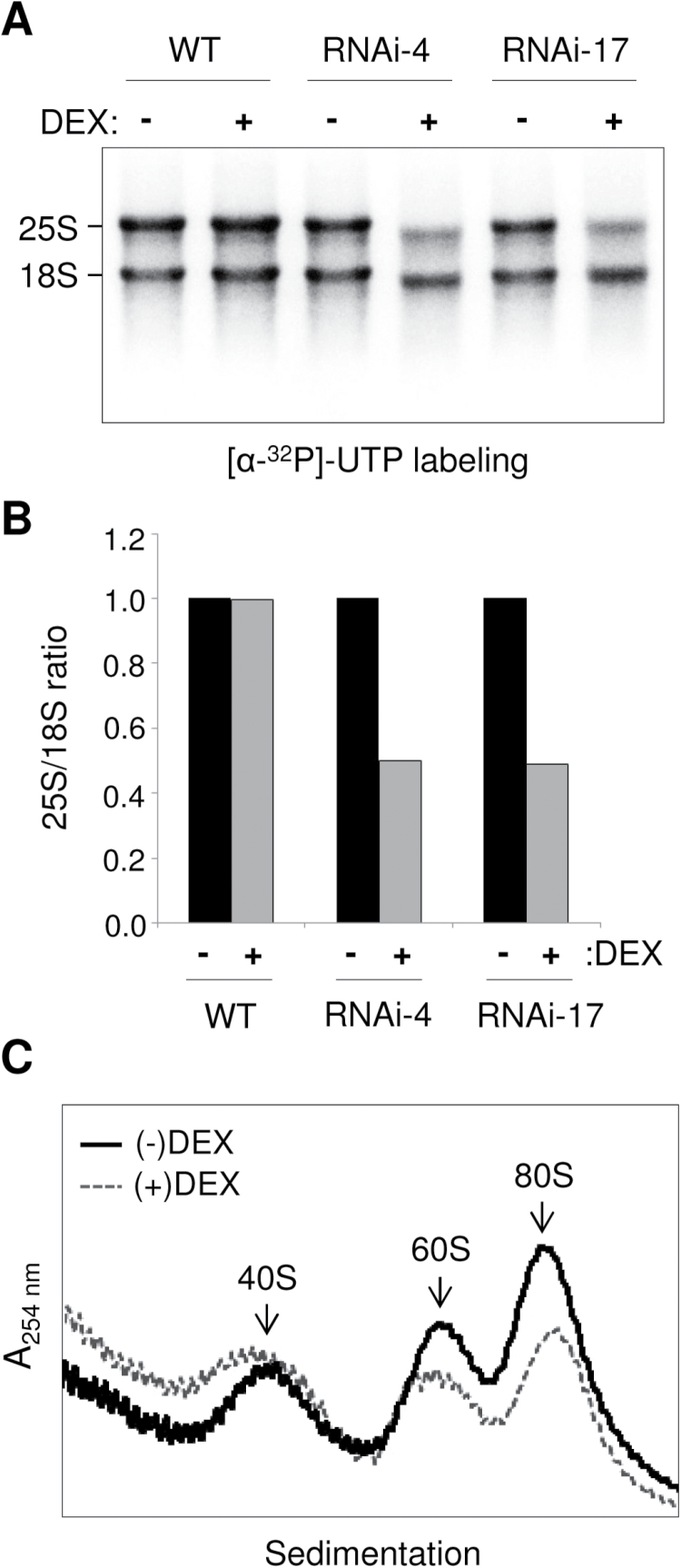
Delayed rRNA processing and ribosome biogenesis in NSN1-deficient *Arabidopsis* plants. Whole seedlings grown for 7–8 d on (–)DEX or (+)DEX media were used for the analyses. (A) Metabolic rRNA labelling. Seedlings were labelled with [α-^32^P]UTP. Total RNA extracted from the seedlings was separated by agarose gel electrophoresis and analysed with a phosphorimager. Newly synthesized mature 25S and 18S rRNAs are marked. (B) Relative 25S/18S rRNA ratio. Band intensities of 25S rRNA and 18S rRNA in the samples shown in (A) are compared. (C) Absorbance profiles of ribosomes at 254nm. Ribosomes were purified from seedlings using ultracentrifugation on a sucrose density gradient.

The effect of NSN1 depletion on nascent rRNA synthesis was examined using *in vivo* [α-^32^P]UTP labelling (Fig. 7A). DEX-inducible *NSN1* RNAi *Arabidopsis* seedlings were grown in media with ethanol (–DEX) or with DEX for 7–8 d. After incorporation of [α-^32^P]UTP into nascent RNA transcripts, total RNA was purified and separated by agarose gel electrophoresis. Two main radioactive bands were detected, which represented newly synthesized 25S and 18S rRNAs. The ratio of 25S rRNA to 18S rRNA was calculated based on the band intensities of rRNAs (Fig. 7B). The 25S/18S rRNA ratios suggest that nascent synthesis of mature 25S rRNA was significantly reduced in DEX-treated *NSN1* RNAi samples (#4 and #17). This result is consistent with delayed 25S rRNA processing in the NSN1-deficient nucleolus.

To investigate a potential role for NSN1 in ribosome biogenesis, ribosome profiling was performed in the *NSN1* RNAi seedlings grown for 7–8 d with or without DEX. Cell extracts were prepared from seedlings, and fractionated with sucrose density ultracentrifugation, and the resulting ribosome profile was measured at 254nm (Fig. 7C). Peak height, detected by *A*_254 nm_, indicated the level of each ribosomal species. Depletion of NSN1 resulted in reduced accumulation of 60S large subunits and 80S monosomes, suggesting delayed assembly of functional 60S subunits. Collectively, depletion of NSN1 caused delayed 25S rRNA maturation and 60S ribosome biogenesis in plant cells.

### Premature senescence phenotypes of NSN1-deficient plants

**Fig. 8.**
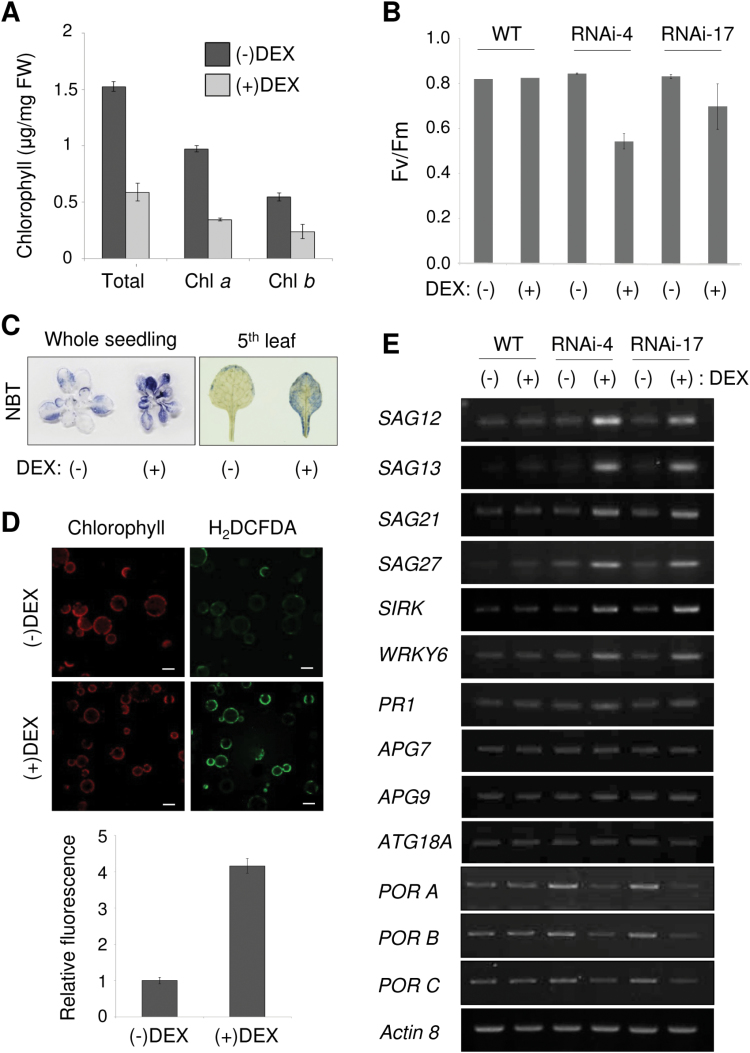
Premature senescence phenotypes of NSN1-deficient *Arabidopsis* plants. The fifth rosette leaf of seedlings grown for 14 d on (–)DEX or (+)DEX media was used for the analyses except in (B). (A) Chlorophyll content in *Arabidopsis* DEX-inducible *NSN1* RNAi lines (RNAi-4) in response to DEX treatment. (B) *F*_v_/*F*_m_ ratio in WT, RNAi-4, and RNAi-17 plants. Plants were grown for 2 weeks in MS medium containing either ethanol or 20 μM DEX, then transferred to soil, and sprayed with ethanol or 20 μM DEX for 3–4 d. (C) Nitro blue tetrazolium (NBT) staining to visualize superoxide production in RNAi-4 lines. (D) H_2_DCFDA staining to visualize H_2_O_2_ production in RNAi-4 lines. After staining, leaf protoplasts were observed under confocal microscopy (top). Relative H_2_DCFDA fluorescence was quantified by confocal microscopy (bottom). Data represent the means ± SD of 110 individual protoplasts. Scale bars = 50 μm. (E) RT–PCR analyses of transcript levels of senescence-related genes in WT, RNAi-4, and RNAi-17 plants. The actin 8 mRNA level was used as the control. (This figure is available in colour at *JXB* online.)

NSN1 deficiency caused growth retardation and gradual leaf yellowing in young *Arabidopsis* plants (Fig. 1E). The premature senescence phenotype of the *Arabidopsis NSN1* RNAi seedlings (Fig. 8) was assessed. DEX-treated RNAi-4 seedlings had less chlorophyll than (–)DEX seedlings (Fig. 8A). This result was similar to that for *NbNSN1* VIGS in *N. benthamiana* (Fig. 1C). The reduced chlorophyll content in DEX-treated seedlings correlated with lower photosynthetic activities as indicated by the optimum quantum yield (*F*_v_/*F*_m_) (Fig. 8B). The *F*_v_/*F*_m_ ratio reflects the maximal photochemical efficiency of photosystem II. The *F*_v_/*F*_m_ ratio in the leaves of (+)DEX RNAi lines was significantly reduced compared with that of (–)DEX controls, suggesting a reduction in functional photosystem II centres (Fig. 8B). Consistent with enhanced senescence of DEX-treated *NSN1* RNAi seedlings, the seedlings also accumulated excessive amounts of reactive oxygen species (ROS) visualized by nitro blue tetrazolium (NBT) (Fig. 8C). NBT reacts with superoxide radicals to form a dark-blue formazan precipitate (Bielski *et al.*, 1980). Excessive ROS formation in NSN1-deficient plants was further visualized by staining with H_2_DCFDA, which emits a green fluorescent signal in the presence of H_2_O_2_, thus indicating oxidative stress in a cell (Fig. 8D, top). Accumulation of green fluorescence in protoplasts from DEX-treated RNAi-4 seedlings was ~4.3-fold greater than in (–)DEX controls, indicating high ROS accumulation (Fig. 8D, bottom).

Leaf senescence-related gene expression was analysed by RT–PCR (Fig. 8E). DEX treatment induced expression of the senescence marker genes *SAG12*, *SAG13*, *SAG21*, and *SAG27* in *NSN1* RNAi seedlings, accompanied by up-regulation of the senescence-induced receptor kinase *SIRK* and the senescence-induced transcription factor *WRKY6*. Expression of the defence marker gene *PR1* and three autophagy-related genes (*APG7*, *APG9*, and *ATG18A*) remained unchanged. In accordance with a decrease in chlorophyll contents, expression of the chlorophyll biosynthetic genes *PORA*, *PORB*, and *PORC* was reduced in *NSN1* RNAi seedlings in response to DEX (Fig. 8E). Collectively, these gene expression changes are in agreement with leaf senescence symptoms observed in *NSN1*-silenced plants.

## Discussion

In this study, it was demonstrated that NSN1 predominantly localized to the nucleolus, primarily through its N-terminal domain. Recombinant NSN1 proteins exhibited GTPase activity and RNA binding activity. NSN1 and its N-terminal domain co-fractionated with 60S ribosome large subunits and 80S monosomes in a sucrose gradient. NSN1 interacted with the ribosome biogenesis factors PES and EBP2, and with several ribosomal proteins, in the nucleolus. NSN1 depletion led to delayed 25S rRNA maturation and 60S ribosome biogenesis, and caused reduced global translation. On an organismal level, NSN1 deficiency caused growth retardation and premature plant senescence. Based on these results, it is proposed that NSN1 plays a role in plant growth and development through its modulation of ribosome biogenesis.

In yeast Nug1, the N-terminal domain associates with pre-60S ribosome particles, binds RNA, and localizes to the nucleolus (Bassler *et al.*, 2006). Similarly, the basic N-terminal domain of NSN1 was targeted to the nucleolus and co-fractionated with the ribosome subunits (Figs 2, 5). The domain directly bound RNA and interacted with the ribosome biogenesis factors PES and EBP2 (Fig. 3; Supplementary Fig. S5 at *JXB* online). These results suggest that the NSN1 N-terminal domain, like that of yeast Nug1, plays a critical role in ribosome association and nucleolar localization by interacting with rRNAs and ribosome biogenesis factors. In human NS, however, both the GTP-binding motifs and the basic N-terminal domain are crucial for nucleolar localization (Tsai and McKay, 2002, 2005). In addition, in the *Caenorhabditis elegans* NS homologue NST-1, deletion of the GTP-binding motifs did not affect the nucleolar distribution of the protein, but abrogated its germline-specific functions (Kudron and Reinke, 2008). The finding that human *NS* cannot complement the null-mutant phenotypes of *Schizosaccharomyces pombe NS* homologue *Grn1* and *C. elegans NST-1* further supports functional divergence among NS homologues (Du *et al.*, 2006; Kudron and Reinke, 2008).

Treatment of human cells with either MPA or a low concentration of actinomycin D, which specifically inhibits rRNA synthesis, causes translocation of a number of nucleolar proteins, including NS, nucleolin, and nucleophosmin, from the nucleolus to the nucleoplasm (Tsai and McKay, 2005; Huang *et al.*, 2008). It has been proposed that human NS dynamically shuttles between the nucleolus and nucleoplasm via a GTP-driven mechanism (Tsai and McKay, 2005). Fluorescence recovery after photobleaching (FRAP) revealed that the N-terminal basic domain and the GTP-binding domain are required for the low affinity and high affinity binding of NS in the nucleolus, respectively, and, furthermore, GTP binding to NS is involved in its long-term residence in the nucleolus. The finding that lowering intracellular GTP levels by MPA treatment causes redistribution of human NS from the nucleolus to the nucleoplasm supports the FRAP results (Tsai and McKay, 2005). Interestingly, nucleophosmin and nucleolin, which shuttle between the nucleolus and nucleoplasm, lack the GTP-binding motifs, despite the fact that nucleophosmin exhibits GTP-dependent nucleolar retention (Finch *et al.*, 1995). More recently, it has been reported that depletion of guanine nucleotide by MPA treatment primarily disrupts pre-rRNA synthesis, similar to the effect of actinomycin D, followed by nucleolar disruption and efflux of nucleolar proteins that may mediate cell cycle arrest and apoptosis (Huang *et al.*, 2008). It was observed that MPA treatment induced translocation of NSN1 from the nucleolus to the nucleoplasm in leaf cells, and the translocation depended on intact GTP-binding motifs (Fig. 2C). Since deletion of the GTP-binding domain did not abolish the nucleolar retention of NSN1 (Fig. 2B), it is possible that the NSN1 mutants (∆GD and ∆G1–3) lacking the GTP-binding motifs may have abnormal protein conformations, which prevent the mutants from moving into the nucleoplasm upon nucleolar stress. It requires further study to illustrate the mechanism of NSN1 translocation upon nuclear stress and the function of the translocated NSN1 in the nucleus.

The YlqF (RbgA) GTPase of *B. subtilis* is essential for late assembly of the 50S ribosome large subunit (Matsuo *et al.*, 2006; Uicker *et al.*, 2006). YlqF specifically associates with the 50S subunit, possibly through interactions with 23S rRNA and RPL25 (Matsuo *et al.*, 2006). YlqF depletion reduced the amounts of 50S subunits and 70S ribosomes, and induced accumulation of the 45S intermediate that lacked L16, L17, and L36 ribosomal proteins (Uicker *et al.*, 2006). These results suggest that YlqF may regulate the structural organization of the assembling 50S subunit to recruit these ribosomal proteins at a late stage of ribosome biogenesis. Among eukaryotic YlqF homologues, yeast Nug1 is closely linked to biogenesis of 60S ribosome subunits: Nug1 is associated with 60S pre-ribosomal particles, and knockdown of *Nug1* reduced the ratio of the 60S subunit to the 40S subunit on sucrose gradients (Bassler *et al.*, 2001, 2006). Affinity purification of TAP-tagged Nug1 co-precipitated pre-60S ribosome subunits that contained Nop7 (PES), Erb1 (BOP1), and other ribosome biogenesis factors, suggesting a direct association of Nug1 with ribosome particles (Bassler *et al.*, 2006). In human HeLa cells, *NS* knockdown delayed rRNA processing, reduced rRNA levels, and reduced protein synthesis (Romanova *et al.*, 2009). Defective rRNA processing was also detected in NS homologue deletion mutants of *S. pombe* and *C. elegans* (Du *et al.*, 2006; Kudron and Reinke, 2008). In this study, delayed 25S rRNA maturation and 60S ribosome biogenesis, and reduced global translation were observed in NSN1-deficient plant cells (Figs 6, 7). These results collectively suggest a conserved role for NS homologues in 60S ribosome biogenesis in eukaryotic cells, reminiscent of prokaryotic RbgA/YlqF/YawG GTPases, which act in 50S ribosome biogenesis. However, these eukaryotic proteins have structural domains that are not present in their prokaryotic counterparts, and appear to interact with different ribosomal proteins (Fig. 4; Matsuo *et al.*, 2006; Romanova *et al.*, 2009). Further study may reveal the action mechanisms of eukaryotic NS homologues in 60S ribosome biogenesis.

BiFC and co-immunoprecipitation analyses indicate that NSN1 interacts with PES and EBP2 in the nucleolus (Fig. 4). PES and EBP2 are essential proteins that are conserved among eukaryotes (Strezoska *et al.*, 2000, 2002; Lapik *et al.*, 2004; Grimm *et al.*, 2006). It was previously reported that plant PES plays an essential role in pre-rRNA processing for maturation of 25S rRNA and biogenesis of the 60S large ribosome subunits (Cho *et al.*, 2013). Yeast EBP2 plays a crucial role in maturation of 25S rRNA and assembly of 60S subunits; EBP2-deficient cells exhibit delayed pre-rRNA processing, primarily affecting 25S rRNA production, and reduced 60S subunit accumulation (Huber *et al.*, 2000; Tsujii *et al.*, 2000). It is known that both PES and EBP2 modulate the late processing steps of pre-rRNAs for the synthesis of 28/25S and 5.8S rRNAs. The 25S rRNA defects observed in NSN1-depleted plant cells and the interactions among NSN1, PES, and EBP2 in the nucleolus suggest that these biogenesis factors are functionally linked to mediate 60S ribosome maturation in plants. As an rRNA-binding protein and a GTPase, NS may create a specific conformation of the pre-rRNA complex for efficient processing. Alternatively, NS may recruit or regulate other ribosome assembly factors such as RNA helicases and rRNA processing factors.

Ribosome biogenesis is a critical regulatory mechanism for cell cycle progression: the amount of ribosomes produced at the end of the G_1_ phase controls the G_1_–S phase transition (Donati *et al.*, 2012). The tumour suppressor p53 has a critical role in cell cycle arrest and/or apoptosis in response to impaired ribosome biogenesis in mammals (Hölzel *et al.*, 2010). Under normal conditions, p53 is made unstable by the action of MDM2, an E3 ubiquitin ligase that targets p53 for proteasomal degradation. Disruption of any key step in ribosome biosynthesis induces nucleolar stress, releasing free ribosomal and nucleolar proteins into the nucleoplasm (Dai *et al.*, 2004, 2006; Zhu *et al.*, 2009). The released ribosomal proteins bind to MDM2 to block its ubiquitinylation of p53, leading to p53 stabilization and subsequent induction of p53-mediated G_1_ cell cycle arrest or apoptosis (Tsai and McKay, 2002; Dai *et al.*, 2008). Recently, p53-independent mechanisms linking nucleolar stress to the cell cycle have been revealed. Nucleolar stress caused by perturbation of ribosome biogenesis resulted in cell proliferation arrest in organisms such as *Drosophila* and yeast, which lack p53 (Donati *et al.*, 2012; James *et al.*, 2014). During ribosomal stress, RpL11 is released from ribosomes and associates with MDM2, disrupting the interaction between the E2F-1 transcription factor and MDM2, which subsequently causes proteasomal degradation of E2F-1 and cell cycle arrest (Donati *et al.*, 2012). Furthermore, mammalian cells with defective p53 exhibited cell cycle arrest in response to ribosomal stress. The p53-independent mechanisms through PIM kinase and the RPL3/SP1/NPM complex have been linked to up-regulation of the cyclin-dependent kinase inhibitors p27^Kip1^ and p21^Cip1^, respectively, resulting in cell cycle arrest (Li *et al.*, 2009; Iadevaia *et al.*, 2010; Russo *et al.*, 2013; James *et al.*, 2014).

Depletion of NS GTPase causes cell cycle arrest and apoptosis in human cells (Ma and Pederson, 2008; Lo and Lu, 2010). NSN1 deficiency resulted in growth retardation and premature senescence in plants (Figs 1, 8). Accumulating data suggest that mammalian NS links the p53 pathway with ribosome biogenesis during cell growth and proliferation (Lo and Lu, 2010). It has been hypothesized that re-localization and subsequent degradation of NS during stress may trigger the release of ribosomal proteins from the nucleolus to the nucleoplasm, implying that NS is a possible sensor of nucleolar stress (Lo and Lu, 2010). Nucleolar stress can be induced by environmental stresses such as heat shock, hypoxia, oxidative stress, and UV irradiation (James *et al.*, 2014). Although plants lack p53 and MDM2, it would be interesting to investigate whether a similar NSN1-mediated sensing mechanism linking nucleolar stress to cell cycle arrest and senescence exists in plants.

## Supplementary data

Supplementary data are available at *JXB* online.

Figure S1. Protein sequence alignment of NSN1 and its homologues.

Figure S2. Expression profiles of *Arabidopsis NSN1* (At3g07050) based on the Genvestigator program (https://www.genevestigator.com/).

Figure S3. Chlorophyll contents.

Figure S4. Nucleolar localization of NSN1.

Figure S5. Subcellular localization of NSN1 mutants.

Figure S6. BiFC analyses.

Figure S7. BiFC between NSN1 and PES mutants.

Figure S8. Immunoblotting with anti-RPL10a antibody to determine cellular levels of RPL10a proteins.

Table S1. PCR primers used in this study

Methods S1. Materials and methods with supplementary references.

Supplementary Data
