# Supplementary material for: The nucleolar GTPase nucleostemin-like 1 plays a role in plant growth and senescence by modulating ribosome biogenesis
Source: J Exp Bot. 2015 Jul 10;66(20):6297–310. doi: 10.1093/jxb/erv337 (PMC4588883; doi:10.1093/jxb/erv337)
Supplement: Supplementary Data [file supp_66_20_6297__index.html]

The nucleolar GTPase nucleostemin-like 1 plays a role in plant growth and senescence by modulating ribosome biogenesis — The nucleolar GTPase nucleostemin-like 1 plays a role in plant growth and senescence by modulating ribosome biogenesis — Supplementary Data 

# The nucleolar GTPase nucleostemin-like 1 plays a role in plant growth and senescence by modulating ribosome biogenesis

## Supplementary Data

Data files

- Supplementary Data - Supplementary Data
