# Supplementary material for: The nucleolar GTPase nucleostemin-like 1 plays a role in plant growth and senescence by modulating ribosome biogenesis
Source: J Exp Bot. 2015 Jul 10;66(20):6297–310. doi: 10.1093/jxb/erv337 (PMC4588883; doi:10.1093/jxb/erv337)
Supplement: Supplementary Data [file supp_erv337_jexbot147488_file001.pdf]

Supplementary Table S1

PCR primers used in this study

Primer name	Sequence	
NbNSN1(F)-F	5’-GGATCCATGGTGAAGAAAAGCAAG-3’	VIGS constructs
NbNSN1(F)-R	5’-GTCGACTTCATTATCCAACTCAACCC-3’	
NbNSN1(N)-F	5’-GGATCCATGGTGAAGAAAAGCAAG-3’	
NbNSN1(N)-R	5’-GTCGACATCTCGAGCATCAAGCAC-3’	
NbNSN1(C)-F	5’-GGATCCTGATGTTGATGCTGCAGC-3’	
NbNSN1(C)-R	5’-GTCGACTTCATTATCCAACTCAACCC-3’	
EBP2(F)-F	5’-GTCGACATGGTTGAGGATGAATTTGCC-3’	
EBP2(F)-R	5’-GGGCCCTCTTTTCGAGCGTTTGTTTT-3’	
EBP2(N)-F	5’-GTCGACATGGTTGAGGATGAATTTGCC-3’	
EBP2(N)-R	5’-GGGCCCTTCTCAGCTAAGAGTCGG-3’	
EBP2(C)-F	5’-GTCGACTAGCTGAGAA GAGGAAG-3’	
EBP2(C)-R	5’-GGGCCCTCTTTTCGAGCGTTTGTTTT-3’	
NSN1-A-F	5’-CCGTAAAGAGAGGGCAAAGA-3’	Realtime qRT-PCR for VIGS
NSN1-A-R	5’-CCGGTGTGGAACTCATCATC-3’	
NSN1-B-F	5’-GTGCCACTAGGAGACAAAATGA-3’	
NSN1-B-R	5’-TTTTTCTGTTTAGTGTTCAACATGC-3’	
NSN1-C-F	5’-ATGTCAAGAAGGATTCTGCCA-3’	
NSN1-C-R	5’-TTTTAAGATGGTCCTATAAGGG-3’	
EBP2-A-F	5’-GTCGACTAGCTGAGAA GAGGAAG-3’	
EBP2-A-R	5’-AATCTAGGCCAGCAGCACCCTC-3’	
EBP2-B-F	5’-ATGGTTGAGGATGAATTTGCC-3’	
EBP2-B-R	5’-TTTCAAGCACACCGTCCC-3’	
NSN1i-sense-F	5’-CTCGAGAGACGGTTAACGAATTCAACCCTG-3’	DEX-inducible RNAi constructs
NSN1i-sense-R	5’-ATCGATAATCTCCGTCCATCAGATCCTCC-3’	
NSN1i-antisense-F	5’-ACTAGTAGACGGTTAACGAATTCAACCCTG-3’	
NSN1i-antisense-R	5’-CTGCAGTAATCTCCGTCCATCAGATCCTCC-3’	
NSN1-F	5’-TTGGAGGTTCTTGATGCC-3’	Realtime qRT-PCR for RNAi
NSN1-R	5’-ATTTCTCAGCAGCCTCTCG-3’	
UBC10-F	5’-ATGGGTCCTTCAGAGAGTCCT-3’	Controls for qRT-PCR
UBC10-R	5’-TGGAACACCTTGGTCCTAAAG-3’	
α-tubulin-F	5’-ATGGCTTGCTGCCTTATGTT-3’	
α-tubulin-R	5’-CACAGCAGCATTGACATCCT-3’	
NSN1(GFP)-F	5’-GGATCCATGGTGAAACGGAGT-3’	GFP-tagging constructs
NSN1(GFP)-R	5’-GGATCCTTCTTCAGGCAAAAGTCC-3’	
NSN1-N(GFP)-F	5’-GGATCCATGGTGAAACGGAGT-3’	
NSN1-N(GFP)-R	5’-GGATCCCAGCAGCAACACAAGAT-3’	
ΔN(GFP)-F	5’-GGATCCAACAAGATTGAGCTTGTTCC-3’	
ΔN(GFP)-R	5’-GGATCCATGGTGAAACGGAGT-3’	
ΔGD(GFP)-F	5’-GGATCCATGGTGAAACGGAGT-3’	
ΔGD(GFP)-R	5’-GGATCCTTCTTCAGGCAAAAGTCC-3’	
NSN1-C(GFP)-F	5’-GGATCCATGGCTTCCATAGCTCTCCG-3’	
NSN1-C(GFP)-R	5’-GGATCCTTCTTCAGGCAAAAGTCC-3’	
ΔG1-3(GFP)-F	5’-GGATCCATGGTGAAACGGAGT-3’	
ΔG1-3(GFP)-R	5’-GGATCCTTCTTCAGGCAAAAGTCC-3’	
NSN1(MBP)-F	5’-GGATCCATGGTGAAACGGAGTA-3’	MBP fusion constructs
NSN1(MBP)-R	5’-GTCGACTTCTTCAGGCAAAAGTCC-3’	
NSN1-N(MBP)-F	5’-GGATCCATGGTGAAACGGAGT-3’	
NSN1-N(MBP)-R	5’-GTCGACCAGCAGCAACACAAGAT-3’	
RBD(MBP)-F	5’-GGATCCGGTAAACTTAAAAAG-3’	
RBD(MBP)-R	5’-GTCGACGATTTTACCTTCATTC-3’	

Supplementary Table S1-continued

PCR primers used in this study

Primer name	Sequence	
NSN1(YFP^{C})-F	5'-GGATCCATGGTGAAACGGAGTAA-3'	BiFC constructs
NSN1(YFP^{C})-F	5'-GGATCCTCATTCTTCAGGCAAA-3'	
PES(YFP^{N})-F	5'-ACTAGTATGCCGAAGCATTACA-3'	
PES(YFP^{N})-R	5'-CCCGGGTGATGATGGTTGAGTAC-3'	
PES(YFP^{C})-F	5'-ACTAGTATGCCGAAGCATTACA-3'	
PES(YFP^{C})-R	5'-CCCGGGTGATGATGGTTGAGTAC-3'	
EBP2(YFP^{N})-F	5'-GGATCCATGTCATTGGAAGAGGA-3'	
EBP2(YFP^{N})-R	5'-CTCGAGCCTCTTCTGCCTCTTGTT-3'	
RPL13(YFP^{N})-F	5'-GGATCCATGGTGTCTGGATCAGGA-3'	
RPL13(YFP^{N})-R	5'-GGATCCGTACTTGACGGGTGCAAG-3'	
RPL14(YFP^{N})-F	5'-GGATCCATGGGTTTTAAGAGATTCGTGG-3'	
RPL14(YFP^{N})-R	5'-GGATCCAGCAGCGATCTCCCTT-3'	
RPS6(YFP^{N})-F	5'-GGATCCATGAAGTTCAACGTTGCG-3'	
RPS6(YFP^{N})-R	5'-GGATCCTTAAGCTGTGACAGAGGG-3'	
RPL11(YFP^{N})-F	5'-GGATCCATGGCGTCTTCTTCTCTATC-3'	
RPL11(YFP^{N})-R	5'-GGATCCTTACAATAAAACTGCTTTC-3'	
PES-Flag-F	5'-GGATCCATGCCGAAGCATTACA-3'	Co-IP constructs
PES-Flag-R	5'-CCATGGGTGATGATGGTTGAGTAC-3'	
NSN1-HA-F	5'-GGATCCATGGTGAAACGGAGTAA-3'	
NSN1-HA-R	5'-GGATCCTCATTCTTCAGGCAAA-3'	
EBP2-Myc-F	5'-GTCGACATGTCATTGGAAGAGGATATAG-3'	
EBP2-Myc-R	5'-GAATTCTTACCTCTTCTGCCTCTTGTTTC-3'	
SAG12-F	5'-TCTCGTCCACTCGACAATGAA-3'	RT-PCR
SAG12-R	5'-AGCTTTCATGGCAAGACCACA-3'	
SAG13-F	5'-CGTGCTCATATCCTCTGCTGC-3'	
SAG13-R	5'-CCGTCAACGCAAATGGTCTG-3'	
SAG21-F	5'-CCAATGCTATCTTCCGACGTG-3'	
SAG21-R	5'-GAACCGGTTTCGGGTCTGTAA-3'	
SAG27-F	5'-TCCTGGCCCTGAAGTAGAAA-3'	
SAG27-R	5'-GTCCCGCAAGAACCTGTCC-3'	
SIRK-F	5'-TCTCTGTTGAAGGAAGCGGTC-3'	
SIRK-R	5'-CAAGCAAGAGCGATCTCTGAC-3'	
WRKY6-F	5'-GGGAAGAGACTTGGGCGTG-3'	
WRKY6-R	5'-GCAACGGATGGTTATGGTTTC-3'	
PR1-F	5'-CACTACACTCAAGTTGTTTGGAG-3''	
PR1-R	5'-CTCGTAATCTCAGCTCTTATTTG-3'	
APG7-F	5'-CCACCTCTCTGTCTGATGATATGC-3'	
APG7-R	5'-GACCAAGCAGCGTGATCTGTGAG-3'	
APG9-F	5'-GGAATCATCCTTATCTTCTTGACTGG-3'	
APG9-R	5'-GTGCTGATCTGAGAGTGGTTGC-3'	
ATG18a-F	5'-GAAACCATTGCCAACCCTAAGG-3'	
ATG18a-R	5'-CCACTGAGCATTTGAAGAGAAGG-3'	
PORA-F	5'-CGTACCCTCTTCCCTCCTTTCC-3'	
PORA-R	5'-CACGAGCTTCTCGCTGACTTCC-3'	
PORB-F	5'-GTTTCACAGGCGTTTCCATG-3'	
PORB-R	5'-GCTTCTCACTGATCTCCCACAC-3'	
PORC-F	5'-CAGTTCAATGATAGATGGAGGAGAG-3'	
PORC-R	5'-GCTGATTCTCAAACGAAGACGAG-3'	
Actin8-F	5'-ATGAAGATTAAGGTCGTGGCA-3'	
Actin8-R	5'-TCCGAGTTTGAAGAGGCTAC-3'	

Supplementary Figure S1

```
NSN1      1 : MVKRSKKSKSKRVTLKQKHKVLKKVKEHHKKKAKDAKKLGLHRKPRVEKDPGIPNDWPFKEQELK :  65
Nb NSN1   1 : MVKKSKKSKSKRVSLKKKHKIIRKVKEHHKKKSKEAKKLGLNKKPKVEKDPGIPNDWPFKEQELK :  65
Os NSN1   1 : MVKKSKKSKSKRVTLRQKHKVLRKVKEHHRKKRKEAKKEGKSHRKKVEKDPGIPNEWPFKEQELK :  65
NS        1 : MKKPKLKKASKRMTCHKRYKIQKKVREHHRKLRKEAKKQG-HKKPR--KDPGVPNSAPFKEALLR :  62
Nug1      1 : --MRVRKRQSRRTSTKLKEGIKKKASAHRKKEKKMAKK-DVTWRSRSKKDPGIESNEPYKAKILE :  62

                                        ΔN
                                        ↓
NSN1     66 : ALEVRRARALEEIEQKKEARKERAKKRKLGLVD--DED---------TKTEGETIEDL------P : 113
Nb NSN1  66 : ALEARRARALEELEQKKAARKERAKKRKLGLIE--DDDDESTPADMTSTKRNNFEEARPNDGST : 128
Os NSN1  66 : ALEARRAQALQELELKKEARKERARKRKLGLIE--DEDIANLASAASAQGSEFAEKDAAKENASL : 128
NS       63 : EAELRKQR-LEELKQQQKLDRQKEQEKKRKLET--NPDI-------KPSNVEPMEKEFGLCKTEN : 117
Nug1     63 : EIEAKKMKDLEERELAKQQRLEARKAAKEQGVDAMDEDMIEDDENGLAALVESAQQAAAEYEGTP : 127

                                                              DAR
NSN1    114 : -------------------------KVVNVRDNSERAFYKELVKVIELSDVILEVLDARDPLGTR : 153
Nb NSN1 129 : -------------------------SFVKHRDNSERAFYKELVKVIDASDVILEVLDARDPLGTR : 168
Os NSN1 129 : -------------------------DVVKSQDHSERAFYKELVKVIEASDVILEVLDARDPLGTR : 168
NS      118 : -------------------------KAKSGKQNSKKLYCQEIKKVIEASDVVLEVLDARDPLGCR : 157
Nug1    128 : SNDADVRDDELDVIDYNIDFYGEDVEGESELEKSRKAYDKIFKSVIDASDVILYVLDARDPESTR : 192

                       G4
NSN1    154 : CTDMERMVMQAGPNKHLVLLLNKIDLVPREAAEKWLMYLREEFPAVAFKCSTQEQRSNLGWK-SS : 217
Nb NSN1 169 : CLDMEKMVMRAGPDKHLVLLLNKIDLIPREAAEKWLKYLREELPTVAFKCSTQEQKSNLGWKPSS : 233
Os NSN1 169 : CIDMEKMVRKADPSKRIVLLLNKIDLVPKESVEKWLTYLREEMPTVAFKCNTQEQRTKLGWK-SS : 232
NS      158 : CPQVEEAIVQSG-QKKLVLILNKSDLVPKENLESWLNYLKKELPTVVERASTKPKDKG---KITK : 218
Nug1    193 : SRKVEEAVLQSQ-GKRLILILNKVDLIPPHVLEQWLNYLKSSFPTIIPLRASSGAVNG-------- : 248

                                                   G1
NSN1    218 : KA--SKPSNMLQTSDCLGADTLIKLLKNYSRSHELKKSITVGIIGLPNVGKSSLINSLK-----R : 275
Nb NSN1 234 : KAGKSKTSNLLQTSDCLGAETLIKLLKNYSRSHEIKKSITVGVIGLPNVGKSSLINSLK-----R : 293
Os NSN1 233 : KI--DKSSNIPQSSDCLGAENLIKLLKNYSRSHELKLAITVGIVGLPNVGKSSLINSLK-----R : 290
NS      219 : RVKAKKNAAPFRSEVCFCKEGLWKLLGGFQEI--CSKAIRVGVIGFPNVGKSSIINSLK-----Q : 276
Nug1    249 : ------TSFNRKLSQTTTASALIESLKTYSNNSNLKRSIVVGVIGYPNVGKSSVINALLARRGGQ : 307

                                   G3
NSN1    276 : AHVVNVGATPGLTRSLQEVHLDKNVKLLDCPGVVM-----LKS-SGNDASIALRNCKRIEKIDDP : 334
Nb NSN1 294 : SHVVNVGATPGLTRSLQEVQLDKNVKLLDCPGVVM-----LRSASEDDASIALRNCKRIEKLDDP : 353
Os NSN1 291 : SRVVNVGSTPGVTRSMQEVQLDKKVKLLDCPGVVM-----LKS-SNSGVSVALRNCKRVEKMEDP : 349
NS      277 : EQMCNVGVSMGLTRSMQVVPLDKQITIIDSPSFIV-------SPLNSSSALALRSPASIEVVK-P : 333
Nug1    308 : SKACPVGNEAGVTTSLREIKIINKLKILDSPGICFPSENKKRSKVEHEAELALLNALPAKHIVDP : 372

NSN1    335 : VSPVKEILKLCPKDMIVI-----IYKIPSFEAVD------DFIYKVATVRGKIKKGGIVDIDAAA : 388
Nb NSN1 354 : VGPVKEILKLCPERMIVI-----IYKVPTFDSVD------DFIQKVATVRGRIKKGGIVDVDAAA : 407
Os NSN1 350 : ISPVKEILDLCPHEKILS-----IYRVPTFTSVD------DFIQKVATIRGKIKKGGIVDVEAAA : 403
NS      334 : MEAASAIISQADARQVVL-----KYTVPGYRNSL------EFFTMLAQRRGMHQKGGIPNVEGAA : 387
Nug1    373 : YPAVLMLVKRLAKSDEMIESFKKLYEIPPIPANIADTFTKHFIIHVARKRGRIGKGGIPNIASAG : 437

NSN1    389 : RIVIHDWNEGKIPYYTMPPKR-DQGGHAESKIVTEIAKDFNIDEVYSGESSFIGSLKTVNEFNPV : 452
Nb NSN1 408 : RIVIHGWNEGKIPYYTLPPTR-NEGEHSEATIVSEIGKEFNIDEVYGSESSIIGSLKSVNDFNPV : 471
Os NSN1 404 : RIVIHDWNEGKIPYFTVPPKR-DAVDDSDAVIISETGKEFNIDEIYKAESSYIGGLKSLEEFRHI : 467
NS      388 : KLIWSEWTGASIAYYCHPPTSWTPPPYFNESIVVDMKSGFNIEELEKNDAQSIRAIKGPHLANSI : 452
Nug1    438 : LSVINDWRDGKILGWVLPNTS-AAASQQDKQNLSTINTGTKQAPIAANESTIVSEWSKEFDLIGL : 501

NSN1    453 : IIPSNGPLNFDETMIEDESKTQTEEEA----EHESDDDESMGGEEEEEAGKTKEKSETGRQNVKL : 513
Nb NSN1 472 : EVPSNRPVNFDENMLEDNLQQSLAENDNSIDKSVSENEDEPMDSREGDAGQTRGKSATRRQNEKL : 536
Os NSN1 468 : EIPSNAPPQIDEEMLEDGKKQNEPAQE--------NHDESMSDANEREGAKTASAST---QNDKL : 521
NS      453 : LFQSSGLTN---GIIEEKDIHEELPKR--------KERKQEEREDDKDSDQETADEEVDENSSGM : 506
Nug1    502 : F--SSLDKAIDASKDQETMME-------------------------------------------- : 520

NSN1    514 : YAAESMLNTKKQKAEKKKRKKAKKAGADEEDLMDGDYDFKVDYRKNKDGEDEEFQIDAKIPMAGL : 578
Nb NSN1 537 : YAEEGMLNTKQKKAEKKRRKKDKPSTAT--DMMDDDYDFKVDYVKKDSAMDDAEDTETTDGSKNN : 599
Os NSN1 522 : YTAEGILDPRKRKAEKKRRKANKFSVLTD---MDADYDFKVDYQMKDALAEEGNDGGDEEPKEAD : 583
NS      507 : FAAEETGEALSEETTAGEQ-STRSFILDKIIEEDDAYDFSTDYV--------------------- : 549
Nug1      - : ----------------------------------------------------------------- :   -

NSN1    579 : LPEE----------- : 582
Nb NSN1 600 : RFELPSGVELDNENH : 614
Os NSN1 584 : PMTGVDDA------- : 591
NS        - : --------------- :   -
Nug1      - : --------------- :   -
```

Supplementary Fig. S1. Protein sequence alignment of NSN1 with its homologs.

Amino acid sequences of *Arabidopsis* NSN1 (At3g07050) and its homologs from *Nicotiana benthamiana* (*Nb* NSN1: NbS00039776g0013), *Oryza sativa* (*Os* NSN1; Os01g0375000), *Homo sapiens* (NS; AAV74413), and *Saccharomyces cerevisiae* (Nug1; P40010) were aligned. The conserved GTPase motifs (G4, G1, and G3) and the "DAR" motif of unknown function are defined according to Matsuo *et al.* (2014) and marked with *overlines*. Motifs G2 and G5 are ill defined in this group of GTPases. The deletion point of NSN1ΔN is marked in red.

Supplementary Figure S2

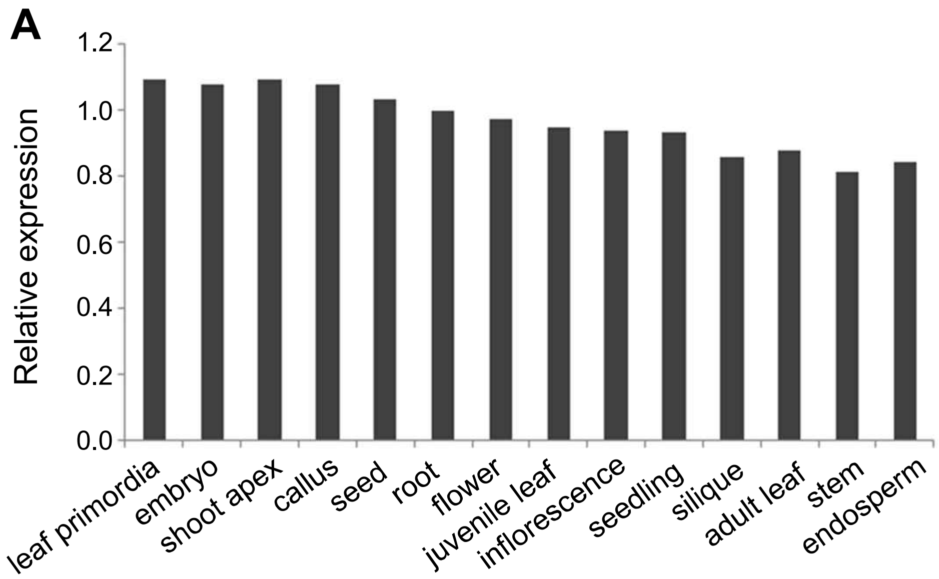

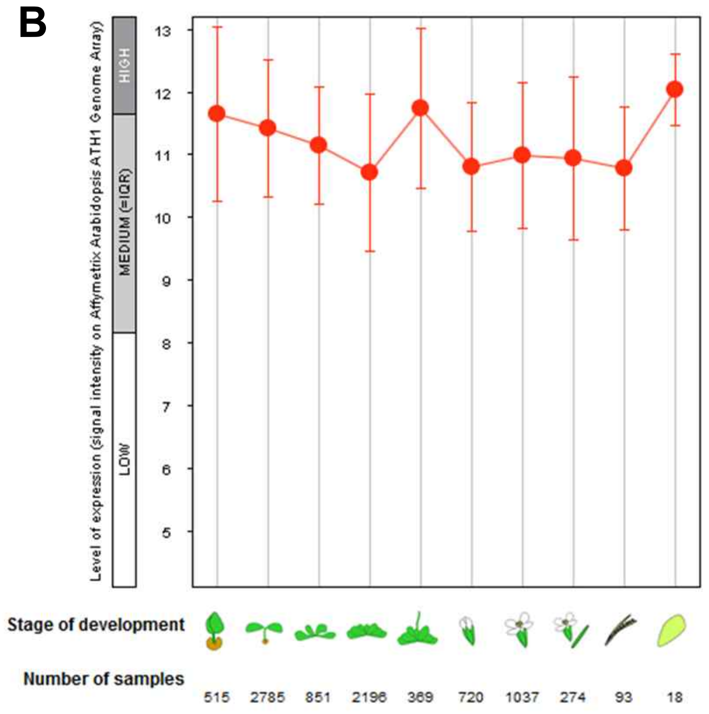

Supplementary Fig. S2. Expression profiles of *Arabidopsis NSN1* (At3g07050) based on the Genevestigator program (https://www.genevestigator.com/).

(A) *NSN1* transcript levels in various tissues relative to the transcript level in roots.

(B) Relative *NSN1* transcript levels in plants at various developmental stages.

Supplementary Figure S3

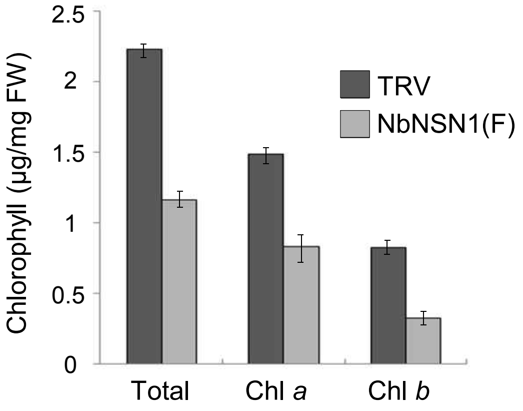

Supplementary Fig. S3. Quantification of total chlorophyll, chlorophyll *a*, and chlorophyll *b* contents in TRV and TRV:NbNSN1(F) leaves at 20 DAI.

Supplementary Figure S4

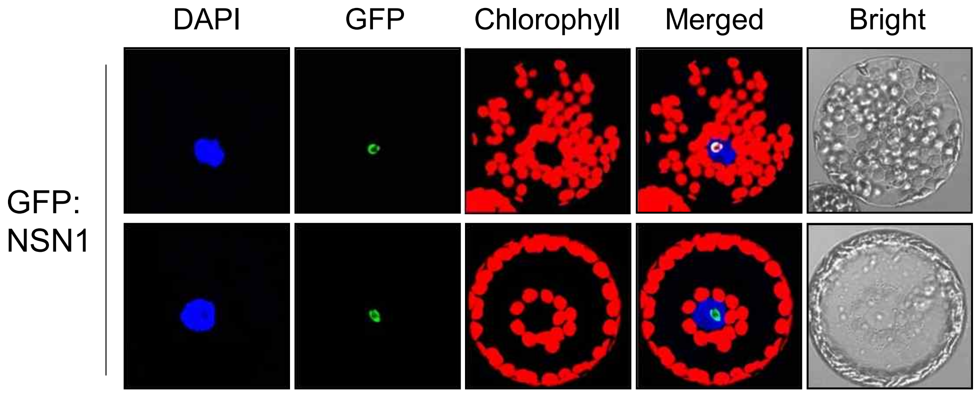

Supplementary Fig. S4. Nucleolar localization of NSN1.

GFP-fused NSN1 was expressed in *N. benthamiana* leaves by agroinfiltration. GFP fluorescence in mesophyll protoplasts prepared from the infiltrated leaves was examined by confocal microscopy. Nuclei were visualized by DAPI staining. Chlorophyll autofluorescence is shown in red.

Supplementary Figure S5

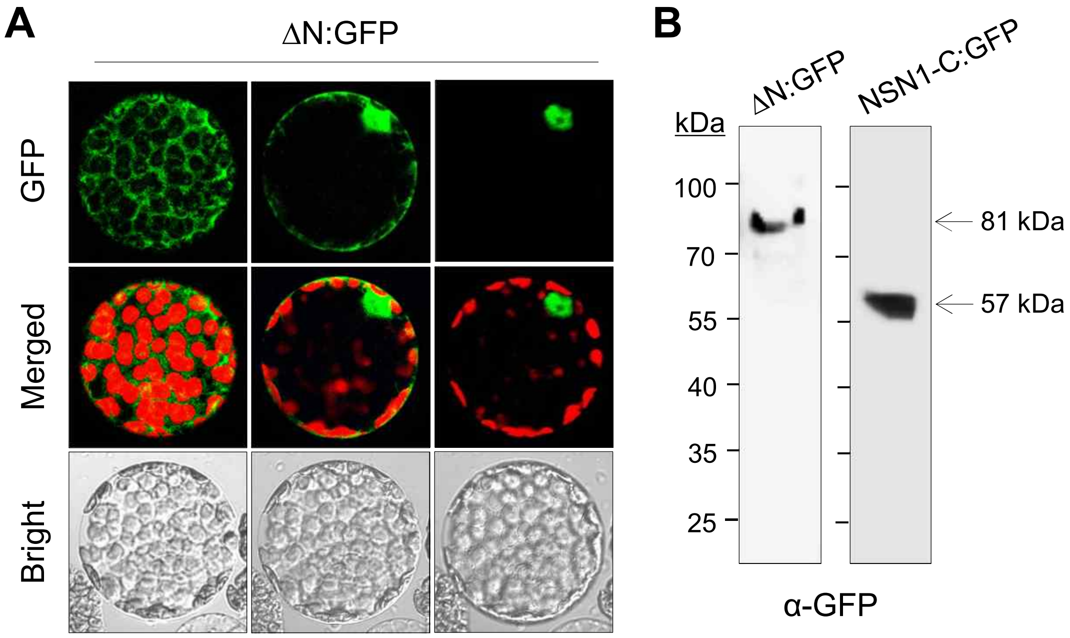

Supplementary Fig. S5. Subcellular localization of NSN1 mutants.

(A) Optical sectioning generated diverse fluorescent images of ΔN:GFP proteins in a single *N. benthamiana* leaf protoplast. Chlorophyll autofluorescence is shown in red.

(B) Expression of ΔN:GFP and NSN1-C:GFP proteins in the infiltrated leaves was determined by immunoblotting with anti-GFP antibody.

Supplementary Figure S6

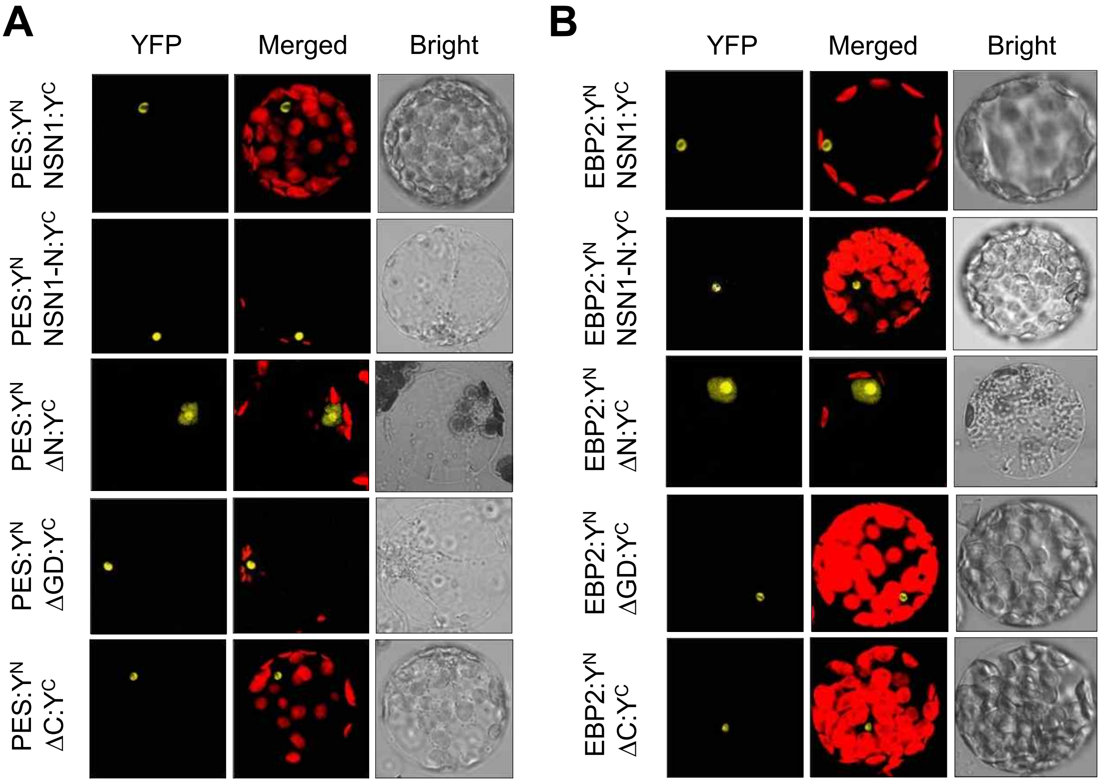

Supplementary Fig. S6. BiFC analyses.

BiFC was performed to visualize the interactions of NSN1 and its deletion mutants with PES (A) and EBP2 (B). YFP^{N} (Y^{N})- and YFP^{C} (Y^{C})-fusion proteins were expressed together in *N. benthamiana* leaves by agroinfiltration. YFP fluorescence in mesophyll protoplasts prepared from the infiltrated leaves was examined by confocal microscopy.

Supplementary Figure S7

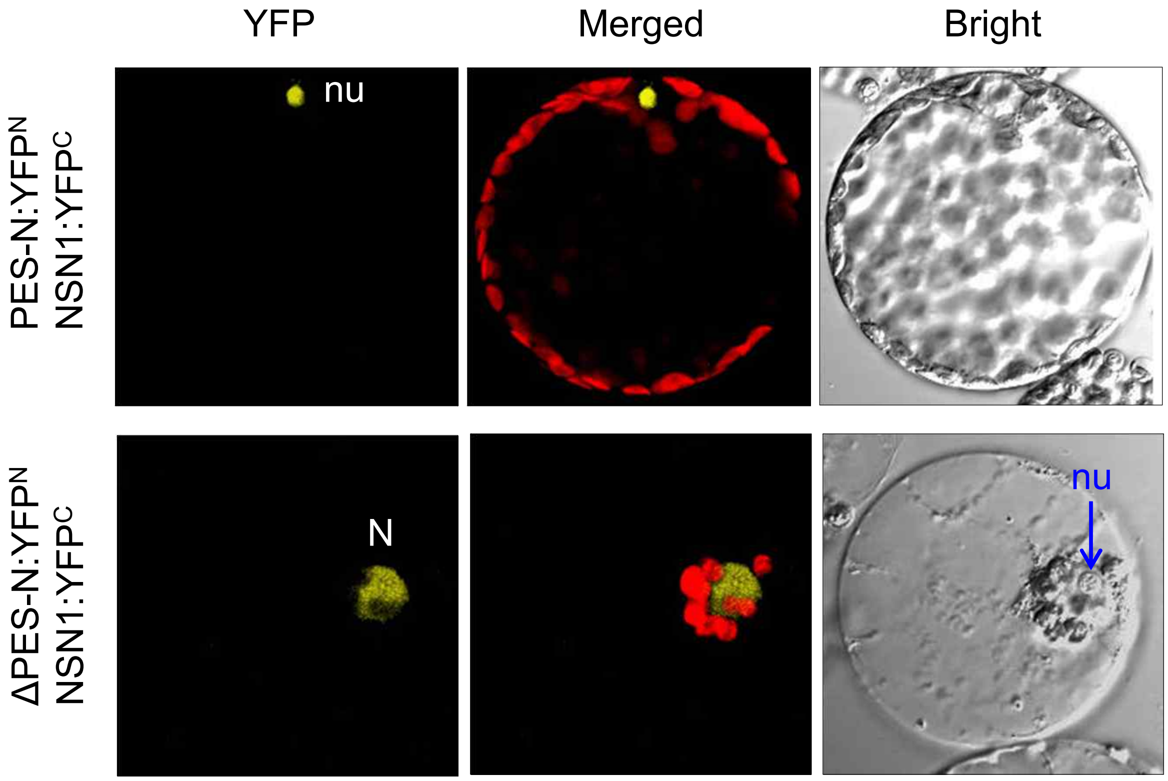

Supplementary Fig. S7. BiFC between NSN1 and PES mutants.

BiFC was performed with NSN1 and the N-terminal domain of PES (PES-N) or the PES mutant lacking the PES-N domain (ΔPES-N). YFP^N- and YFP^C-fusion proteins were expressed together in *N. benthamiana* leaves by agroinfiltration. YFP fluorescence in mesophyll protoplasts prepared from the infiltrated leaves was examined by confocal microscopy. *N*, nucleus; *nu*, nucleolus.

Supplementary Figure S8

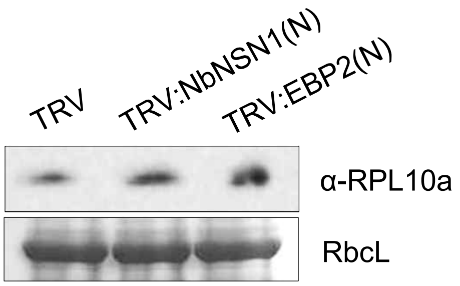

Supplementary Fig. S8. Immunoblotting with anti-RPL10a antibody to determine cellular levels of RPL10a proteins.

The 4th leaf above the infiltrated leaf of the VIGS plants at 14 DAI were used for the analysis. Coomassie blue-stained Rubisco large subunits (RbcL) were used as loading control.

Supplementary Methods S1

Virus-induced gene silencing (VIGS)

Functional genomics using Tobacco Rattle Virus (TRV)-based virus-induced gene silencing was carried out in *Nicotiana benthamiana* as described (Ahn *et al.*, 2011; Cho *et al.* 2013). Various cDNA fragments of *N. benthamiana NSN1* and *EBP2* were polymerase chain reaction (PCR)-amplified and cloned into the pTV00 vector containing part of the TRV genome (Cho *et al.*, 2013). The recombinant pTV00 plasmids and pBINTRA6 vector containing RNA1, which is required for virus replication, were separately transformed into *Agrobacterium tumefaciens* GV3101. The third leaf of *N. benthamiana* (3-week-old plants) was pressure-infiltrated with the *Agrobacterium* suspension. The fourth leaf above the infiltrated leaf was used for real-time quantitative reverse transcription (RT)-PCR to detect gene silencing.

Generation of dexamethasone (DEX)-inducible NSN1 *RNAi lines in* Arabidopsis

A 318-bp *NSN1* cDNA fragment was PCR amplified using NSN1-sense(F)/(R) primers containing XhoI and ClaI sites (5'-agacggttaacgaattcaaccctg-3' and 5'-aatctccgtccatca gatcctcc-3') for the sense construct and NSN1-antisense(F)/(R) primers containing SpeI and PstI sites (5'-agacggttaacgaattcaaccctg-3' and 5'-aatctccgtccatcagatcctcc-3') for the antisense construct. Using these constructs, DEX-inducible *NSN1* RNAi transgenic *Arabidopsis* lines (ecotype Columbia-0) were generated as described (Ahn *et al.*, 2011). Transgenic plants were selected on growth media containing hygromycin (30 mg/L). For induction of RNAi, dexamethasone (Sigma) was added to the medium to a final concentration of 10 or 20 μM in ethanol (0.033%) and tween 20 (0.01 % w/v) from 30 mM stock solution.

Agrobacterium-mediated transient expression

Agroinfiltration was carried out as described previously (Voinnet *et al.*, 2003). Agrobacterial cultures (*Agrobacterium tumefaciens* GV3101 strain) containing various constructs fused to the CaMV35S promoter were adjusted to OD_{600}=0.6 in MES buffer (10 mM MES, pH 7.5, 10 mM $MgSO_4$). The suspension was incubated with acetosyringone for 2–3h at a final concentration of 150 μM, and infiltrated into leaves of wild-type *N. benthamiana* plants. In all experiments, *Agrobacterium* C58C1 carrying the 35S:p19 construct (Voinnet *et al.*, 2003) was co-infiltrated to achieve maximum levels of protein expression. Expressed proteins

were analyzed at 48h post-infiltration.

Real-time quantitative RT-PCR

Real-time quantitative PCR was carried out using gene-specific primers as described previously (Cho *et al.*, 2013).

Bimolecular fluorescence complementation (BiFC)

The cDNAs of *EBP2*, *PES*, and various ribosomal protein genes of *Arabidopsis* were PCR-amplified and cloned into the pSPYNE vector containing the N-terminal region of YFP (YFP^N; amino acid residues 1-155). Similarly, the coding region of *NSN1* and *PES* were cloned into pSPYCE vector containing the C-terminal region of YFP (YFP^C; residues 156–239). Various combinations of these pSPYNE and pSPYCE fusion constructs were agroinfiltrated together into the leaves of 3-week-old *N.benthamiana* plants as described (Cho *et al.*, 2013). After 48 h, protoplasts were generated and YFP signal was detected using a confocal laser scanning microsope (Zeiss LSM510).

Measurement of in vivo H_2O_2 levels

NBT staining was carried out as described previously (Lee *et al.*, 2013). H_2DCFDA staining was carried out as described previously (Ahn *et al.*, 2011).

Immunoblotting

Protein extracts (30µg) were subjected to SDS-PAGE and immunoblotting as described (Ahn *et al.*, 2011; Cho *et al.*, 2013). Immunoblotting was performed using mouse monoclonal antibodies against the HA tag (Applied Biological Materials), Myc tag (ABM), Flag tag (Sigma), GFP (Clontech), and RPL10a (Santa Cruz Biotechnology). The membranes were then treated with horseradish peroxidase-conjugated goat anti-mouse IgG antibodies (1:10,000; Invitrogen). Signals were detected on X-ray film (Kodak) using an ECL chemiluminescence kit (ELPIS-Biotech, Inc.).

Co-immunoprecipitation

HA-tagged NSN1 and Myc-tagged EBP2, or HA-tagged NSN1 and Flag-tagged AtPES were co-expressed in *N. benthamiana* leaves by agroinfiltration. After 48-h incubation, leaf extracts were prepared and co-immunoprecipitation was performed according to Ahn *et al.*

(2011). After SDS-PAGE, western blotting was carried out using the monoclonal anti-HA antibody (1:2500; ABM), anti-Flag antibody (1:10,000; Sigma), and anti-Myc antibody (1:10,000; ABM).

Purification of recombinant proteins

To purify recombinant proteins of NSN1 and its mutants for GTPase assays, the corresponding *NSN1* cDNA fragments were PCR-amplified and cloned into the pMAL^{TM}c2 vector (New England Biolabs). The MBP fusion proteins were purified using amylose resin following the manufacturer's instructions (New England Biolabs). Purified NSN1 protein and its variants were concentrated using Amicon Ultra Centrifugal Filters (Millipore). To purify MBP:NSN1-N and MBP:RBD for RNA-binding assays, the *NSN1* cDNA fragments corresponding to amino acid residues 1−174 and 374-400, respectively, were PCR-amplified and cloned into the pMAL^{TM}c2 vector, and the recombinant proteins were purified as described above.

GTPase assay

The turnover rate (k_{cat}) of recombinant proteins of NSN1 was measured as described previously (Im *et al.*, 2011; Jeon *et al.*, 2014). A reaction mixture containing 3 μM recombinant proteins and 1 mM GTP in GTPase assay buffer (20 mM Hepes, pH 8, 1 mM $MgCl_2$, 0.5 mM DTT, and 1 mM NaN_3) was incubated at room temperature for 18 h. The released phosphate was quantified using the Biomol green reagent (Biomol Research Laboratories) according to the manufacturer’s protocol. The catalytic constant was derived from the equation $k_{cat} = V_{max}/C_{recombinant\ protein}$.

Pulse-amplitude-modulation (PAM) fluorometry

Arabidopsis plants were grown for 2 weeks in MS medium containing either ethanol or 20 μM DEX, then transferred to soil, and sprayed with ethanol or 20 μM DEX for 3-4 days. The plants were incubated in the dark for 4 h, and then the fluorescence analysis was performed with the 5th rosette leaf. The Fv/Fm ratio was determined as described (Kramer *et al.*, 2004). Chlorophyll *a* fluorescence traces were measured using a FMS1 fluorometer (Hansatech instruments, Norfolk, England). F_m and F_0 are the maximum levels of fluorescence before and after the steady state during the actinic illumination, respectively. Fs is the steady-state fluorescence level. The intensities of saturating pulse and actinic lights were more than 5,000

μmol photons / m^2·s and 600 μmol photons / m^2·s, respectively. The quantum yield of photosystem II-mediated photosynthesis (Φ_{II}) is defined as $\Phi_{II} = \frac{F_s - F_m'}{F_m'}$.

Statistical analyses

Two-tailed Student's *t*-tests were performed using the Minitab 16 program (Minitab Inc.; http://www.minitab.com/en-KR/default.aspx) to investigate the statistical differences between the responses of the samples. Significant differences between control and other samples were indicated by one ($P \leq 0.05$) or two ($P \leq 0.01$) asterisks.

Supplementary References

Jeon Y, Ahn CS, Jung HJ, Kang H, Park GT, Choi Y, Hwang J, Pai H-S. 2014. DER containing two consecutive GTP-binding domains plays an essential role in chloroplast ribosomal RNA processing and ribosome biogenesis in higher plants. *Journal of Experimental Botany* **65,** 117-130.

Kramer DM, Johnson G, Kiirats O, Edwards GE. 2004. New fluorescence parameters for the determination of QA redox state and excitation energy fluxes. *Photosynthetic Research* **79,** 209–218.

Lee JY, Lee HS, Song JY, Jung YJ, Reinbothe S, Park YI, Lee SY, Pai HS. 2013. Cell growth defect factor1/chaperone-like protein of POR1 plays a role in stabilization of light-dependent protochlorophyllide oxidoreductase in *Nicotiana benthamiana* and *Arabidopsis*. *The Plant Cell* **25,** 3944-3960.

Voinnet O, Rivas S, Mestre P, Baulcombe D. 2003. An enhanced transient expression system in plants based on suppression of gene silencing by the p19 protein of tomato bushy stunt virus. *The Plant Journal* **33,** 949–956.
